# The genetics of fibromyalgia and its relationships to psychiatric and medical traits

**DOI:** 10.1038/s41467-026-75256-6

**Published:** 2026-07-28

**Authors:** Uri Bright, Sarah Beck, Daniel F. Levey, Joseph D. Deak, The VA Million Veteran Program, J. Michael Gaziano, J. Michael Gaziano, Murray B. Stein, Joel Gelernter

**Affiliations:** 1 Department of Psychiatry, Yale School of Medicine, New Haven, CT USA; 2 Veterans Affairs Connecticut Healthcare System, West Haven, CT USA; 3 Million Veteran Program (MVP) Coordinating Center, VA Boston Healthcare System, Boston, MA USA; 4 Department of Medicine, Brigham and Women’s Hospital, Boston, MA USA; 5 Department of Medicine, Harvard Medical School, Boston, MA USA; 6 VA San Diego Healthcare System, San Diego, CA USA; 7 Department of Psychiatry and School of Public Health, University of California, San Diego, CA USA; 8 Departments of Genetics and of Neuroscience, Yale School of Medicine, New Haven, CT USA

**Keywords:** Genome-wide association studies, Quality of life, Human behaviour

## Abstract

We explore the genetic mechanisms underlying fibromyalgia, a chronic heritable syndrome. We conduct genome-wide association studies (GWAS) of fibromyalgia in European, African, and Latin American ancestry subjects, combining data from several cohorts (85,139 cases; 1,642,433 controls). We also conduct a multi-trait analysis of GWAS (MTAG), leveraging pain GWAS to enhance power for fibromyalgia analyses. We apply a series of methods to analyze genetic association between fibromyalgia and psychological and physiological phenotypes. We find 10 genomic loci that are associated with fibromyalgia in European ancestry subjects, one in African, 12 cross-ancestry, and 45 in the European ancestry MTAG; most of these were previously associated with pain, cognitive function, autoimmune response, or general health measures. We show a moderate negative genetic correlation between fibromyalgia and physical activity, and strong genetic correlations with chronic pain, post-traumatic stress disorder, and depression (r_g_≥0.69). Genomic structural equation modeling places fibromyalgia in the context of psychiatric, medical, and lifestyle phenotypes, mostly as pain- and autoimmune-related trait. Local genetic correlations and genetic causality point to neuronal mechanisms that provide a strong basis for some of the main characteristics of fibromyalgia and its comorbidities. These findings provide potential targets for future studies to improve diagnosis and treatment of fibromyalgia.

## Abstract (web summary)

The authors conduct genome-wide association studies of fibromyalgia in individuals of European, African, and Latin American ancestries. The findings provide potential targets for future studies to improve diagnosis and treatment of this syndrome.

## Introduction

Fibromyalgia is a chronic disorder characterized by nociplastic widespread pain, muscle stiffness, sleep disturbances, fatigue, depression, and anxiety. The syndromic pain is induced by altered nociception, without evident association to injuries, lesions or diseases, and is characterized by central sensitization - amplified pain response to noxious or non-noxious stimuli^1,2^. The point prevalence of fibromyalgia in the world population is 2.7%, with females making up 75% of cases^2^. There are no pathognomonic tests (e.g., blood-based or imaging biomarkers), and its diagnosis is thus based on identifying symptoms that are associated with the disease and exclusion of alternative explanations^1^.

Fibromyalgia is influenced by genetic risk factors^3^: the risk of fibromyalgia among relatives of subjects who suffer from the disorder is eight times higher than in the general population^4^, and it may reach to 13-fold in siblings^5^. Genome-wide association studies (GWAS) have revealed dozens of genes associated with chronic pain and other pain-related disorders^6^. Two fibromyalgia-specific GWAS have been published to date; both used relatively small sample sizes and found no significant loci in European (EUR) or African (AFR) populations^7,8^. A study that examined the genetic basis of fibromyalgia as part of a long list of pain disorders also failed to find any significant single nucleotide polymorphism (SNP) specifically related to fibromyalgia^9^. A GWAS of chronic widespread musculoskeletal pain, a symptom of fibromyalgia, reported three genome-wide associations in *RNF123*, *ATP2C1* and *COMT*^10^.

A phenome-wide association study (pheWAS) revealed significant associations between fibromyalgia and 304 phenotypes (out of 1740 phecodes tested); the strongest of these associations were with pain phenotypes (e.g., chronic pain, joint pain), traits directly related to pain (e.g., osteoarthrosis, spondylosis, migraine), autoimmune disease (erythematosus lupus) and psychiatric disorders (e.g., depression)^8^. Indeed, there is a strong association between fibromyalgia and psychiatric disorders: the lifetime prevalences of depression, anxiety, post-traumatic stress disorder (PTSD) and attention-deficit/hyperactivity disorder (ADHD) among fibromyalgia patients are reported to be 74%, 60%, 57% and 45%, respectively^11–13^.

The therapeutic approach typically includes a combination of psychotherapy and pharmacotherapy (sometimes off-label) and lifestyle changes^2^. The former approaches may involve guided imagery, cognitive behavioral therapy, and group psychotherapy^14,15^, in addition to the introduction or increased frequency of aerobic physical exercise, a first-line treatment for the disorder. There are three FDA-approved medications: duloxetine and milnacipran – both of which are serotonin-norepinephrine reuptake inhibitors (SNRIs) – and pregabalin; the efficacy of opioids is quite limited^2,16^. Several small studies suggest that cannabis may be helpful to alleviate pain in fibromyalgia patients^17^. These treatments are variably effective; new therapeutic approaches are needed.

Currently, it is unclear whether fibromyalgia can be prevented, and the available treatments are often unsatisfactory^18^. In this study, we aimed to discover genetic mechanisms that may identify novel treatment targets and open new paths for research regarding possible ways to alleviate symptoms of this disorder, that impairs the quality of life of so many people worldwide. Further, we hope to contribute to better prediction of who is at risk for the development of fibromyalgia which could lead to discoveries relevant to prevention. To these aims, we conducted a meta-analyzed GWAS in individuals of EUR, AFR, and Latin American (AMR) ancestries, as well as cross-ancestry and sex-stratified analyses, based on several different large samples including All of Us (AoU)^19^, the Million Veteran Program (MVP)^20^, the UK Biobank (UKBB)^21^, and Finngen^22^ biobanks. We also conducted post-GWAS analyses to assess gene-based associations, transcriptome-wide associations, global and local genetic correlations, and genetic causality between fibromyalgia and a list of autoimmune diseases, psychiatric traits and general health phenotypes that are assumed to be associated with fibromyalgia^1,2,23^. Finally, we conducted genomic structural equation modeling (gSEM), to understand the genomic relationships between fibromyalgia and other psychiatric, autoimmune, general health and pain-related traits.

In this work, we show that fibromyalgia is a complex genetic trait, affected by multiple genomic loci and is associated with a variety of pain-related traits, as well as with psychiatric phenotypes, autoimmune response, and general health. In this context, gSEM analysis places fibromyalgia mostly as a pain and autoimmune-related trait. Our results provide novel insights into the genetic architecture of fibromyalgia.

## Results

### Initial GWAS analyses for phenotype definition

In each of the cohorts we analyzed in this study (AoU, MVP, UKBB), we conducted a separate GWAS for every phenotype definition of fibromyalgia (stringent ICD-10, less-stringent ICD-10, self-report), and then calculated the heritability and the genetic correlations between them (Suppl. Data 1). Within AoU and MVP, we found significant genetic correlations between all the different phenotypes, with r_g_ ~ =1.00. Within UKBB, the genetic correlation between the two defined phenotypes was not significant, likely due to relatively small sample sizes. We therefore tested the genetic correlation between the UKBB phenotypes and their closest equivalents in AoU (self-report in UKBB vs self-report in AoU, and stringent ICD-10 in UKBB vs stringent ICD-10 in AoU) and found a nominally significant effect of r_g_ = 0.77 between the self-report traits, and a significant effect for the stringent ICD-10 traits, with r_g_ = 1.07 (Suppl. Data 2). In view of these results, we combined all the phenotypic variations and assembled inclusive fibromyalgia phenotypes in all cohorts (Table 1).

**Table 1:** Demographics

Ancestry	Sex*	Cohort	Measure	Cases	Controls	Total	Effective**	% Cases
EUR	All Subjects	AoU	SR + ICD	11,878	208,531	220,409	44,952	5.39%
		MVP	ICD	41,847	384,192	426,039	150,947	9.82%
		UKBB	SR + ICD	5460	427,444	432,904	21,565	1.26%
		Finngen	ICD-10	3623	357,549	361,172	14,347	1.00%
		**Meta**		62,808	1,377,716	1,440,524	240,278	4.36%
	Females	AoU	SR + ICD	10,775	120,767	131,542	39,570	8.19%
		MVP	ICD	7970	22,751	30,721	23,609	25.94%
		UKBB	SR + ICD	4464	234,875	239,339	17,523	1.87%
		**Meta**		23,209	378,393	401,602	87,471	5.78%
	Males	AoU	SR + ICD	1113	87,915	89,028	4396	1.25%
		MVP	ICD	33,877	361,441	395,318	123,896	8.57%
		UKBB	SR + ICD	782	204,850	205,632	3116	0.38%
		**Meta**		35,772	654,206	689,978	135,670	5.18%
AFR	All Subjects	AoU	SR + ICD	2295	70,616	72,911	8891	3.15%
		MVP	ICD	13,468	94,580	108,048	47,157	12.46%
		**Meta**		15,763	165,196	180,959	57,560	8.71%
	Females	AoU	SR + ICD	2189	40,271	42,460	8305	5.16%
		MVP	ICD	3465	11,153	14,618	10,575	23.70%
		**Meta**		5654	51,424	57,078	20,376	9.91%
	Males	AoU	SR + ICD	108	30,431	30,539	430	0.35%
		MVP	ICD	10,003	83,427	93,430	35,728	10.71%
		**Meta**		10,111	113,858	123,969	37,145	8.16%
AMR	All Subjects	AoU	SR + ICD	2517	67,184	69,701	9704	3.61%
		MVP	ICD	4051	32,337	36,388	14,400	11.13%
		**Meta**		6568	99,521	106,089	24,645	6.19%
	Females	AoU	SR + ICD	2356	43,104	45,460	8935	5.18%
		MVP	ICD	823	2607	3430	2502	23.99%
		**Meta**		3179	45,711	48,890	11,889	6.50%
	Males	AoU	SR + ICD	163	24,149	24,312	648	0.67%
		MVP	ICD	3228	29,730	32,958	11,647	9.79%
		**Meta**		3391	53,879	57,270	12,761	5.92%

Females and males sample sizes do not necessarily sum up to be exactly as the All Subjects figures, due to kinship removal that was done separately for each analysis. **Effective sample size = 4/(1/nCases + 1/nControls) [*SR* self-report; *ICD* ICD-10 code, *AoU* All of Us version 8, *MVP* Million Veterans Program, *Meta* meta-analysis, *UKBB* UK Biobank, *EUR* European, *AFR* African, *AMR* Latin American].

### Genome-Wide Association Studies (GWAS): main analyses

**Fig. 1:**
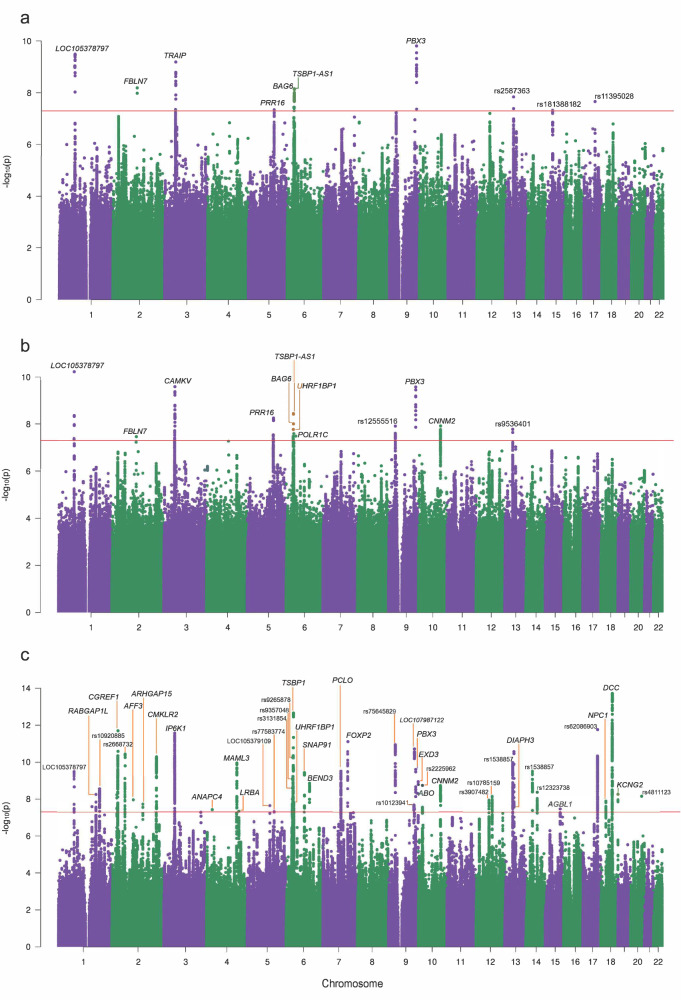
Main analysis. GWAS meta-analysis of fibromyalgia in (**a**) EUR population (n_total_ = 1,440,524, n_eff_ = 240,278), and (**b**) cross-ancestry (EUR-AFR-AMR; n_total_ = 1,727,572, n_eff_ = 323,773). (**c**) MTAG of fibromyalgia leveraging pain data (n_eff_ = 240,278). Significant variants that are located within or near a gene are annotated according to the relevant gene; significant variants which are not located in the proximity of a gene are annotated with their rsID. Association tests were performed using a logistic regression model adjusted for age, sex, and the first ten genetic PCs as covariates. All tests were two-sided. Genome-wide significance was defined at *p* < 5 × 10^−^^8^.

We conducted GWAS of fibromyalgia in three EUR cohorts. For this analysis we used an inclusive fibromyalgia phenotype (see previous section), defined by stringent ICD-10 code, less-stringent ICD-10 code and/or self-report in AoU, by stringent ICD-10 code and less-stringent ICD-10 code in MVP and by stringent ICD-10 code and self-report in UKBB. We found one lead variant in each, all different. In an EUR meta-analysis (n_effective_ = 240,278), which included fibromyalgia summary statistics of the aforementioned AoU, MVP and UKBB analyses, plus summary statistics of stringent ICD-10 definition of fibromyalgia from Finngen (which did not have any significant results on its own), we discovered ten independent lead SNPs: LOC105378797*rs1993709, FBLN7*rs72831629, TRAIP*rs71080556, PRR16*rs56405820, BAG6*rs2242656, TSBP1-AS1*rs9279546, PBX3*rs6478712, rs2587363, rs181388182 and rs11395028 (Table 2, Fig. 1a). For AFR and AMR ancestries, GWAS analyses of fibromyalgia – conducted using an inclusive fibromyalgia phenotype – were possible in two cohorts – AoU and MVP. In AFR, there were no significant regions in AoU and one in MVP - POLR1C*rs186798404 – which survived in the AFR meta-analysis (Suppl. Fig. 2). In AMR there were no significant effects in any of the analyses (Suppl. Fig. 3). A cross-ancestry meta-analysis, which included the three ancestral meta-analyses, revealed 12 significant lead variants: LOC105378797*rs10889947, FBLN7*rs72831629, CAMKV*rs2681780, PRR16*rs190161089, BAG6*rs2242656, TSBP1-AS1*rs9279546, UHRF1BP1*rs16894959, POLR1C*rs186798404, rs12555516, rs10819064, CNNM2*rs75970938 and rs9536401 (Table 2, Fig. 1b). In all the meta-analyses, heterogeneity was not detected for any of the significant SNPs (HetPVal > 0.05; see also Suppl. Fig. 10). Regional Manhattan plots of all our lead SNPs in the various meta-analyses are presented in Suppl. Figs. 11–33. We also conducted sex-stratified analyses, with no significant results for males in any of the individual and meta-analyzed AFR and AMR cohorts. In EUR, in UKBB we found one GWS locus in SUCLG2-DT*rs10539712, which did not survive the meta-analysis. In females, in EUR we found one significant lead SNP in UKBB: CRYBG3*rs188327717; one in AoU: rs1030125981; and none in MVP. In the EUR meta-analysis we found three lead SNPs: IP6K1*rs763622663, PRR16*rs62381083 and PCLO*rs73167394. We did not find any female-specific effects in AFR and AMR. (Table 2, Suppl. Figs. 4–9). The LD between TRAIP*rs71080556, CAMKV*rs2681780, and IP6K1*rs763622663, which were GWS in three different analyses, was relatively high (r^2^ > 0.6; rs71080556:rs2681780, r^2^ = 0.62, d’ = 0.8; rs71080556:rs763622663, r^2^ = 0.95, d’ = 0.98; rs763622663:rs2681780, r^2^ = 0.64, d’ = 0.81).

**Table 2:** Lead SNPs in GWAS meta-analyses of fibromyalgia

Anc.	Sex	Cohort	rsID	Chr	Pos (GRCh38)	EA	Beta	SE	*P*	Gene
EUR	All	AoU	rs112902269	3	49,824,991	CA	0.078	0.014	1.21E-08	*-*
		MVP	rs6478712	9	125,752,329	C	0.045	0.008	5.66E-09	*PBX3*
		UKBB	rs188327717	3	97,935,433	T	0.112	0.018	2.87E-10	*CRYBG3*
		**Meta**	rs1993709	1	72,372,846	A	−0.086	0.014	3.24E-10	*LOC105378797*
			rs72831629	2	112,175,548	T	−0.068	0.012	6.45E-09	*FBLN7*
			rs71080556	3	49,828,612	G	−0.074	0.012	6.44E-10	*TRAIP*
			rs56405820	5	120,795,518	A	0.035	0.007	4.55E-08	*PRR16*
			rs2242656	6	31,646,325	T	0.043	0.008	1.56E-08	*BAG6*
			rs9279546	6	32,264,129	CT	−0.043	0.007	6.95E-09	*TSBP1-AS1*
			rs6478712	9	125,752,329	T	−0.040	0.006	1.54E-10	*PBX3*
			rs2587363	13	53,341,798	A	−0.057	0.010	1.45E-08	*-*
			rs181388182	15	47,119,288	T	0.037	0.007	4.78E-08	*-*
			rs11395028	17	52,208,371	CT	0.066	0.012	2.18E-08	*-*
	F	AoU	rs1030125981	3	49,824,985	CA	0.081	0.014	1.33E-08	*-*
		UKBB	rs188327717	3	97,935,433	T	0.124	0.019	3.48E-09	*CRYBG3*
		**Meta**	rs763622663	3	49,758,958	A	−0.077	0.013	1.37E-09	*IP6K1*
			rs62381083	5	120,794,269	T	−0.062	0.011	1.22E-08	*PRR16*
			rs73167394	7	82,797,010	A	0.060	0.011	2.66E-08	*PCLO*
	M	UKBB	rs10539712	3	67,842,251	CTT	0.538	0.096	2.33E-08	*SUCLG2-DT*
AFR	All	MVP	rs186798404	6	44,338,731	T	0.778	0.141	3.27E-08	*POLR1C*
		**Meta**	rs186798404	6	44,338,731	T	0.778	0.141	3.27E-08	*POLR1C*
Cross-Anc.	All	**Meta**	rs10889947	1	72,362,538	T	−0.065	0.010	5.90E-11	*LOC105378797*
			rs72831629	2	112,175,548	T	−0.062	0.011	3.43E-08	*FBLN7*
			rs2681780	3	49,860,397	T	0.061	0.010	2.54E-10	*CAMKV*
			rs190161089	5	120,651,192	A	−0.070	0.012	5.50E-09	*PRR16*
			rs2242656	6	31,646,325	T	0.035	0.006	1.67E-08	*BAG6*
			rs9279546	6	32,264,129	CT	−0.041	0.007	3.54E-09	*TSBP1-AS1*
			rs16894959	6	34,857,885	T	−0.038	0.007	2.56E-08	*UHRF1BP1*
			rs186798404	6	44,338,731	T	0.778	0.141	3.26E-08	*POLR1C*
			rs12555516	9	31,234,019	C	0.033	0.006	1.20E-08	*-*
			rs10819064	9	125,737,677	T	0.038	0.006	2.64E-10	*-*
			rs75970938	10	103,033,891	T	0.056	0.010	1.20E-08	*CNNM2*
			rs9536401	13	53,343,730	T	0.054	0.010	1.66E-08	*-*

Fibromyalgia was defined by stringent ICD-10 code, less-stringent ICD-10 code and/or self-report. SNPs were assigned by FUMA to the nearest gene based on physical distance ( ± 10 kb from gene boundaries). *Chr* chromosome, *Pos* GRCh38-based position, *EA* effect allele, *AoU* All of Us version 8, *MVP* Million Veterans Program, *UKBB* UK Biobank, *Meta* meta-analysis, *EUR* European, *AFR* African, *AMR* Latin American, *M* males, *F* females, *Anc* ancestry. Association tests were performed using a logistic regression model adjusted for age, sex, and the first ten genetic PCs as covariates. All tests were two-sided. Genome-wide significance was defined at *p* < 5 × 10^−8^.

### Gene-based and gene set analyses

Using MAGMA gene-based analysis we found 148 genes significantly associated with fibromyalgia in EUR. The strongest effects were for *PCLO*, *RNF123* and *DCC* (Suppl. Data 3). In AFR, we found one gene associated with fibromyalgia: *TEX22* (Suppl. Data 4). In AMR, we found no significant genes associated with fibromyalgia (Suppl. Data 5). Cross-ancestry, we found 38 genes significantly associated with fibromyalgia (Suppl. Data 6).

### Heritability estimates and genetic correlations

We used LD score regression (LDSC) to calculate the heritability estimates (h^2^) of the individual and meta-analyzed EUR cohorts, and found that the SNP heritability of fibromyalgia in the EUR meta-analysis is 6.9% (h^2^ = 0.069 ± 0.004) (Supplementary Data 7). We also calculated inter-cohort (i.e., AoU, MVP, UKBB, Finngen) genetic correlations, and found significant values for all the pairs tested, with r_g_ values ranging between 0.72 ( ± 0.1) and 0.95 ( ± 0.1) (Supplementary Data 8). We repeated these tests for the sex-stratified analyses (Supplementary Data 7, 8). The genetic correlation between fibromyalgia in males vs females was r_g_ = 0.73 ( ± 0.064; *p* = 3.7 x 10^−30^). We then calculated the genetic correlations between the EUR meta-analysis of fibromyalgia and 27 traits which are associated with cognitive function, physical activity, substance use, psychiatric disorders, autoimmune, cardiovascular, neurodegeneration, and general health measures. Except for the genetic correlations with Parkinson’s disease (PD) and Alzheimer’s disease (AD), all the r_g_ values were statistically significant. The strongest negative genetic correlation was with physical activity (r_g_ = −0.5 ± 0.027). The strongest positive genetic correlations were with chronic pain (r_g_ = 0.8 ± 0.023), PTSD (r_g_ = 0.72 ± 0.026) and depression (r_g_ = 0.69 ± 0.021), while the next five were of the PTSD subphenotypes hyperarousal (r_g_ = 0.68 ± 0.032), avoidance (r_g_ = 0.63 ± 0.034) and re-experiencing (r_g_ = 0.62 ± 0.032), migraine (r_g_ = 0.58 ± 0.034), and ADHD (rg = 0.57 ± 0.029) (Supplementary Data 9, Fig. 2). For male-only and female-only analyses, the genetic correlations with all 27 traits were in the same direction as in the main analysis, and all the values that were significant in the main analysis were significant also in the female-only analysis. All but six correlations in the male-only analysis were significant after Bonferroni correction (Supplementary Data 9, Fig. 2). We also repeated the genetic correlation calculation for all 27 traits using the fibromyalgia MTAG, with almost identical and all-significant results (Supplementary Fig. 34).

**Fig. 2:**
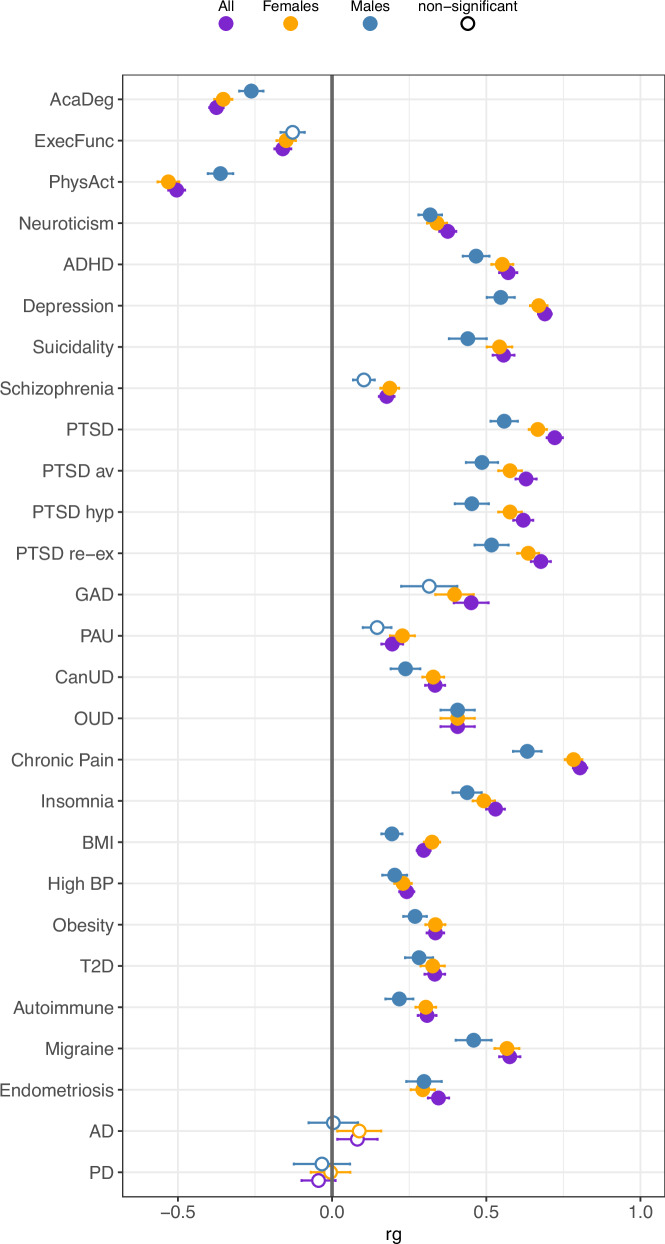
Genetic Correlations. Genetic correlations between fibromyalgia and 27 traits in EUR (sample size for each trait is provided in Supplementary Data 9) in all subjects (purple), in females (orange), and in males (blue), tested using two-sided LD score regression (*p*-value threshold for significance after Bonferroni correction: *p* = 5×10^−4^). The same sets of summary statistics were used for all the analyses (all except endometriosis included subjects of both sexes). Non-significant values are marked with empty circles. Error bars represent standard error. AcaDeg academic degree, ExecFunc executive functioning, PhysAct physical activity, ADHD attention-deficit/hyperactivity disorder, PTSD post-traumatic stress disorder, av avoidance, hyp hyperarousal, re-ex re-experiencing, GAD generalized anxiety disorder, PAU problematic alcohol use, CanUD cannabis use disorder, OUD opioid use disorder, BMI body mass index, BP blood pressure, T2D type 2 diabetes, AD Alzheimer’s disease PD Parkinson’s disease. Source data are provided as a Source Data file.

### Multi-Trait Analysis of GWAS (MTAG)

**Fig. 3:**
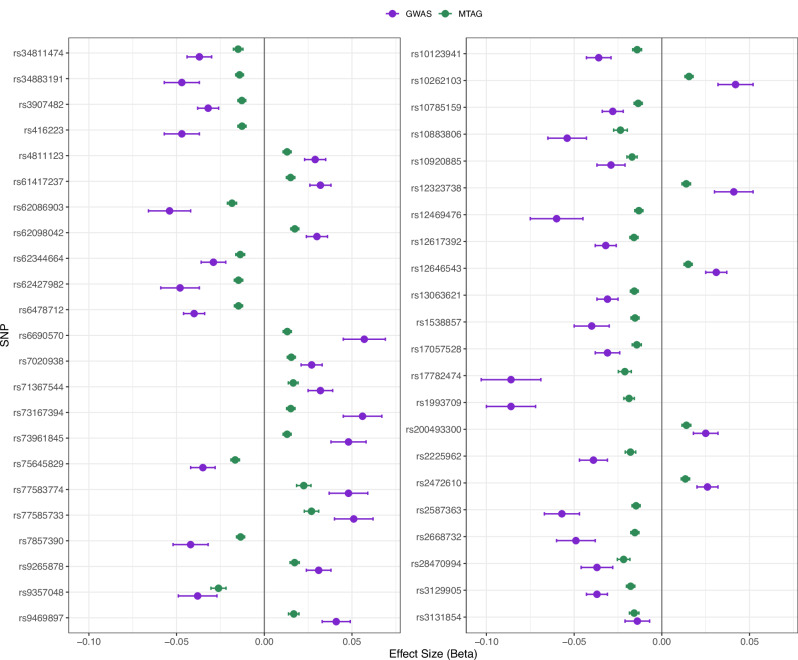
MTAG-GWAS comparison. Effect size of all the significant SNPs in the fibromyalgia MTAG analysis (n_eff_ = 1,016,498), and comparison with their effect size in the EUR fibromyalgia GWAS meta-analysis (n_eff_ = 240,278). Source data are provided as a Source Data file. Error bars represent standard error.

MTAG was conducted using pain summary statistics^22^ to increase the available information for the fibromyalgia GWAS results. The pain phenotype was a generalized trait that included diagnoses of limb, back, neck, head and abdominal pain. The genetic correlation between fibromyalgia and pain was r_g_ = 0.72 ( ± 0.026; *p* = 1.54 x 10^−169^). SNP-based heritability of the MTAG was 8% (h^2^ = 0.08 ± 0.003) (Suppl. Data 7), and the genetic correlation between the fibromyalgia GWAS and MTAG (based on it but including also fibromyalgia variance retrieved from the pain GWAS) was r_g_ = 0.93 ( ± 0.007) (Supplementary Data 8). The genetic correlations between the fibromyalgia MTAG and a variety of other traits were almost identical to the genetic correlations with the fibromyalgia GWAS (Supplementary Data 9, Supplementary Fig. 34), indicating that the “enhanced” GWAS behaved similarly to the original GWAS in terms of its genetic relationships. The EUR fibromyalgia MTAG resulted in 45 independent significant lead SNPs, an increase of 35 compared to the EUR fibromyalgia GWAS. Of the ten GWS loci in the fibromyalgia GWAS, seven were retained in MTAG filtering (that is, only seven of the ten original independent SNPs could be studied in the MTAG context; two were filtered out because they did not exist in the pain summary statistics, and one because it was multiallelic). The effects of all 45 lead SNPs in the MTAG were in the same direction as in the EUR fibromyalgia main analysis. Effect size estimates were smaller for all but one SNP in the MTAG analysis compared to the GWAS, yet with smaller SE values and higher statistical significance (Fig. 3, Supplementary Data 10). The strongest effects in the MTAG were for rs62098042*DCC (*p* = 1.95 x 10^−14^) and rs3129905*TSBP1 (*p* = 2.22 x 10^−13^) (Table 3, Fig. 1c). We also conducted MTAG using both pain and leave-one-out (LOO)-summary statistics of MDD together^24^, excluding for the MDD data cohorts that overlap with those we used for the EUR fibromyalgia GWAS. The genetic correlation between fibromyalgia and the LOO-MDD data was r_g_ = 0.63 ( ± 0.025; *p* = 1.05 x 10^−141^), and between pain and LOO-MDD was r_g_ = 0.56 ( ± 0.021; *p* = 3.18 x 10^−150^). MTAG resulted in 94 independent significant lead SNPs, 34 of which were significant in the pain-leveraged MTAG (Supplementary Data 11).

**Table 3:** Lead SNPs in fibromyalgia MTAG in subjects of EUR genetic ancestry

rsID	Chr	Pos (GRCh38)	EA	Beta	SE	*P*	Gene
rs1993709	1	72372846	A	−0.019	0.003	3.26E-10	*LOC105378797*
rs6690570	1	174239038	T	0.013	0.002	5.54E-09	*RABGAP1L*
rs10920885	1	191058818	T	−0.017	0.003	2.72E-09	*-*
rs12469476	2	22536001	A	−0.013	0.002	5.77E-09	*LINC01884*
rs12617392	2	27113959	A	−0.016	0.002	2.01E-12	*CGREF1*
rs2668732	2	60259880	A	−0.015	0.002	3.72E-11	*-*
rs17782474	2	99649474	A	−0.021	0.004	1.09E-08	*AFF3*
rs73961845	2	143721414	A	0.013	0.002	1.86E-08	*ARHGAP15*
rs77585733	2	206212000	A	0.027	0.004	5.20E-11	*CMKLR2*
rs13063621	3	49784192	T	−0.016	0.002	2.66E-12	*IP6K1*
rs34811474	4	25407216	A	−0.015	0.003	3.79E-08	*ANAPC4*
rs12646543	4	140015960	T	0.015	0.002	1.15E-10	*MAML3*
rs62344664	4	150457762	A	−0.014	0.003	4.58E-08	*LRBA*
rs416223	5	104655775	A	−0.013	0.002	2.26E-08	*LOC105379109*
rs77583774	5	124623861	A	0.023	0.004	4.80E-08	*-*
rs9357048	6	27779263	A	−0.026	0.004	7.97E-10	*-*
rs3131854	6	29644108	T	−0.016	0.003	9.64E-09	*-*
rs9265878	6	31345256	A	0.017	0.003	1.70E-11	*-*
rs3129905	6	32326786	T	−0.018	0.002	2.22E-13	*TSBP1*
rs9469897	6	34823688	A	0.017	0.003	1.50E-08	*UHRF1BP1*
rs61417237	6	83614440	A	0.015	0.002	3.78E-10	*SNAP91*
rs62427982	6	107115962	T	−0.015	0.002	1.40E-09	*BEND3*
rs73167394	7	82797010	A	0.015	0.002	3.06E-10	*PCLO*
rs10262103	7	114451789	A	0.015	0.002	7.88E-12	*FOXP2*
rs75645829	9	31228381	A	−0.017	0.002	1.14E-11	*-*
rs10123941	9	117755884	T	−0.014	0.003	2.10E-08	*-*
rs7020938	9	119902930	A	0.015	0.002	1.96E-11	*LOC107987122*
rs6478712	9	125752329	T	−0.015	0.002	2.54E-10	*PBX3*
rs7857390	9	133253159	A	−0.013	0.002	4.93E-09	*ABO*
rs28470994	9	137387536	T	−0.022	0.004	1.17E-09	*EXD3*
rs2225962	10	19034984	A	−0.018	0.003	1.84E-09	*-*
rs10883806	10	102953319	T	−0.024	0.004	1.90E-09	*CNNM2*
rs3907482	12	60160296	A	−0.013	0.002	1.13E-08	*-*
rs10785159	12	74946958	T	−0.013	0.002	7.11E-09	*-*
rs2587363	13	53341798	A	−0.015	0.002	1.07E-10	*-*
rs1538857	13	58901289	T	−0.015	0.002	2.74E-11	*-*
rs17057528	13	60026176	T	−0.014	0.003	2.46E-08	*DIAPH3*
rs34883191	14	46792330	T	−0.014	0.002	3.12E-10	*-*
rs12323738	14	69033926	T	0.014	0.002	4.39E-09	*-*
rs200493300	15	86382193	T	0.014	0.003	3.37E-08	*AGBL1*
rs62086903	17	68019890	T	−0.018	0.003	1.77E-12	*-*
rs2472610	18	23558901	T	0.013	0.002	1.28E-08	*NPC1*
rs62098042	18	53376190	A	0.017	0.002	1.95E-14	*DCC*
rs71367544	18	79814374	T	0.016	0.003	5.57E-09	*KCNG2*
rs4811123	20	51098956	T	0.013	0.002	7.16E-09	*-*

SNPs were assigned by FUMA to the nearest gene based on physical distance ( ± 10 kb from gene boundaries). *Chr* chromosome, *Pos* GRCh38-based position, *EA* effect allele. Association tests were performed using a logistic regression model adjusted for age, sex, and the first ten genetic PCs as covariates. All tests were two-sided. Genome-wide significance was defined at *p* < 5×10^−^^8^.

### Mendelian Randomization (MR)

**Fig. 4:**
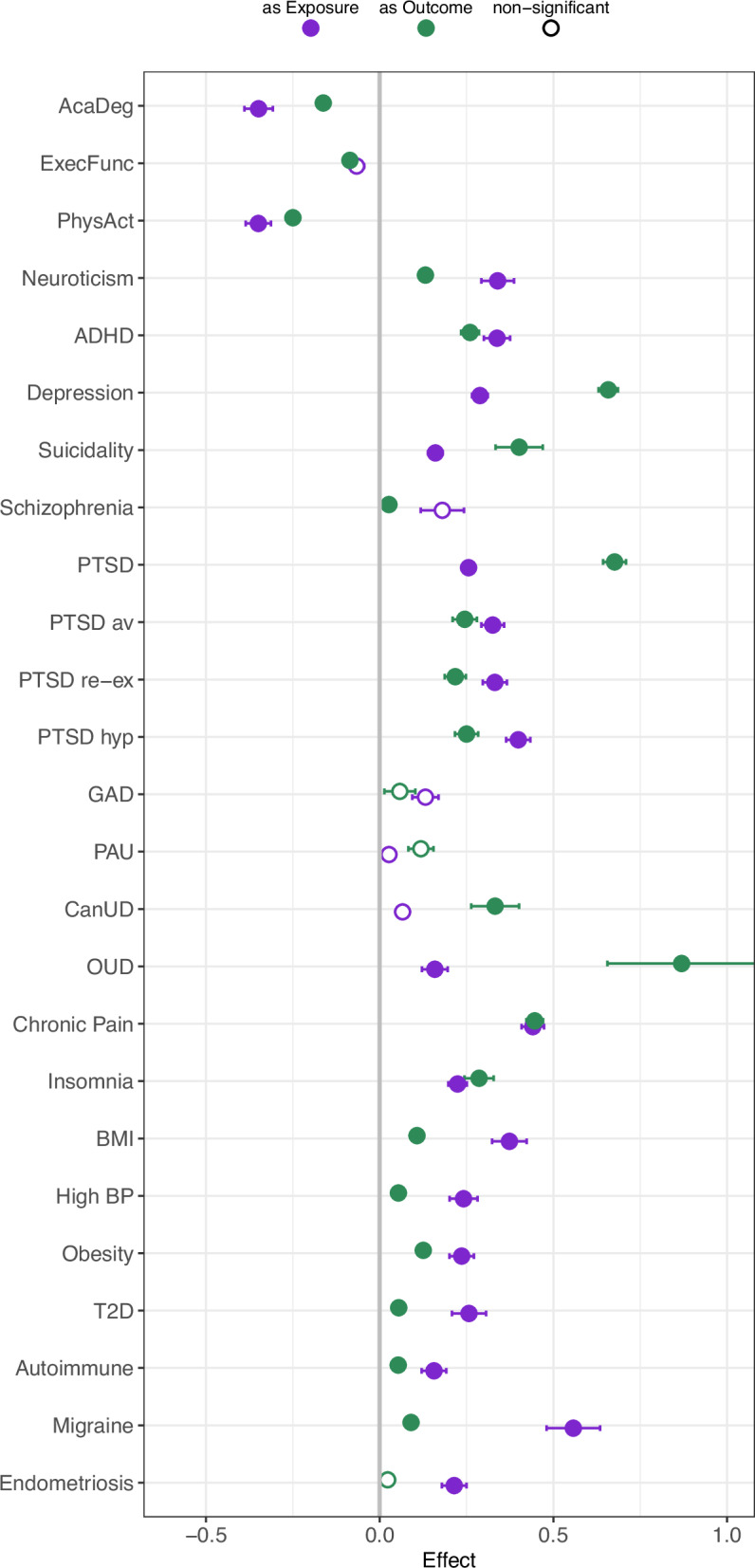
Causal Genetic Correlations. Causal genetic correlations between fibromyalgia (as exposure and as outcome) and 25 traits in EUR (sample size for each trait is provided in Supplementary Data 9), tested using Mendelian randomization with MRlap (inverse-variance MR, *p*-value threshold for significance after Bonferroni correction: *p* = 1.47 × 10^−4^). Non-significant values are marked with empty circles. Error bars represent standard error. AcaDeg academic degree, ExecFunc executive functioning, PhysAct physical activity, ADHD attention-deficit/hyperactivity disorder, PTSD post-traumatic stress disorder, av avoidance, hyp hyperarousal, re-ex re-experiencing, GAD generalized anxiety disorder, PAU problematic alcohol use, CanUD cannabis use disorder, OUD opioid use disorder, BMI body mass index, BP blood pressure, T2D type 2 diabetes. Source data are provided as a Source Data file.

We used MRlap to assess the inferred causality between genetic liability to fibromyalgia and genetic liability to traits that had a significant effect in the LDSC analyses. We used a *p*-value threshold of 1 x 10^−5^ to define genetic instruments. We found significant causal effects of fibromyalgia (as exposure) on 20 of the 25 traits we examined and a significant causal effect of 22 traits on fibromyalgia (as outcome). All the effects in this test were in the same directions as in the genetic correlation analyses. Problematic alcohol use (PAU) and generalized anxiety disorder (GAD) were the only traits which had no causal correlation with fibromyalgia in either direction (Fig. 4, Supplementary Data 12, 13) (an MR analysis with an instrument *p*-value threshold of 1 x 10^−8^ was conducted as well; results are presented in Supplementary Data 14, 15). While MRlap was used to account for sample overlap, additional methods were implemented to assess the robustness of the causal estimates under different assumptions, using two-sample MR^25^. All significant effects detected by MRlap were in the same direction when using different MR methods (Supplementary Data 16). We also tested horizontal pleiotropy between traits, and found potential horizontal pleiotropy, with low but statistically significant MR-Egger intercept values, between fibromyalgia and chronic pain, neuroticism, high blood pressure (BP), and migraine (Supplementary Data 17). Therefore, MR results regarding the causality between fibromyalgia and these traits should be taken with caution.

### Local Analysis of covariant Association (LAVA)

We used LAVA to calculate the local genetic correlations between fibromyalgia and the 27 traits that were used to calculate r_g_ in EUR (see above). Seventeen traits had at least one local genetic correlation with fibromyalgia – including all three PTSD sub-phenotypes – most prominently PTSD (six loci) and chronic pain (5 loci). In total, there were 43 local genetic correlations considering all pairs, involving 31 different genetic loci. The region between chr2: 59,024,862:60,547,931 correlated five different traits – chronic pain, depression, PTSD and two of its subdimensions (hyperarousal, re-experiencing) – with fibromyalgia. The region between chr11:112,755,447:113,889,019 correlated four different traits – cannabis use disorder (CanUD), executive functioning, neuroticism and PTSD – with fibromyalgia (Suppl. Data 18).

### Cross-ancestry genetic correlations

Using Popcorn^26^, we found that the heritability estimates of fibromyalgia in AFR and AMR subjects were not statistically significant (*p* = 0.18 and *p* = 0.58, respectively). Therefore, genetic correlations between fibromyalgia within those populations and other traits in EUR could not be calculated.

### Transcriptome-Wide Association Study (TWAS)

We used TWAS to evaluate predicted changes in differential gene expression in EUR. We identified four independent associated genes in four different tissues: *DPYSL5* with a positive enrichment in the cerebellum, and *DAG1*, *GPX1* and *PBX3* with negative enrichment in the tibial artery, skeletal muscles and pancreas, respectively (Supplementary Data 19).

### Summary-based Mendelian Randomization (SMR)

One variant, PBX3*rs6478712, remained significant after Bonferroni correction and after excluding genes that failed the HEIDI test (p_HEIDI < 0.05, indicating that the association was likely driven by two distinct variants in LD rather than a single shared causal variant). This variant had a significant negative effect in two tissues: non-sun-exposed skin (*β* = −0.157 ± 0.03) and tibial artery (*β* = −0.131 ± 0.025), suggesting that the effect of this variant on *PBX3* expression in these tissues may be causal with respect to its effect on fibromyalgia (Supplementary Data 20).

### Genomic Structural Equation Modeling (gSEM)

**Fig. 5:**
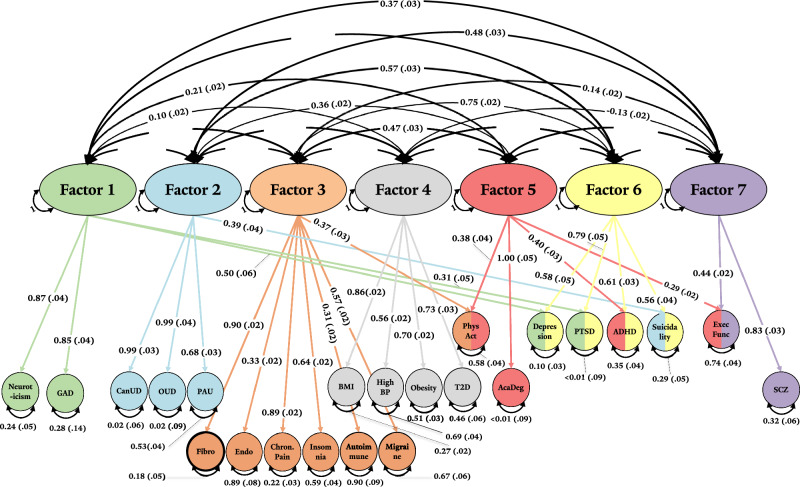
Genomic structural equation modeling (gSEM). The overarching genetic relationship between fibromyalgia and 22 traits of interest. Fibromyalgia loaded on factor 3 AcaDeg academic degree, ExecFunc executive functioning, PhysAct physical activity, ADHD attention-deficit/hyperactivity disorder, PTSD post-traumatic stress disorder, av avoidance, hyp hyperarousal, re-ex re-experiencing, GAD generalized anxiety disorder, PAU problematic alcohol use, CanUD cannabis use disorder, OUD opioid use disorder, BMI body mass index, BP blood pressure, T2D type 2 diabetes, SCZ schizophrenia]. Source data are provided as a Source Data file.

We performed gSEM to examine the overarching genetic relationships between fibromyalgia and 22 traits of interest that had demonstrated significant genetic correlations with fibromyalgia. EFA suggested a seven-factor model fit the data best, explaining 68.9% of cumulative variance. Factor 1 (SS loading: 3.09) explained 13.4% of the variance, factor 2 (SS loading: 3.03) explained 13.2%, factor 3 (SS loading: 2.75) explained 12%, factor 4 (SS loading: 2.32) explained 10%, factor 5 (SS loading: 1.81) explained 8%, factor 6 (SS loading: 1.77) explained 8%, and factor 7 (SS loading: 1.09) explained 5% of the overall variance. Traits with EFA loadings > 0.25 were evaluated on the respective factors in CFA. CFA suggested that the seven-factor model fit the data well via traditional fit indices including a comparative fit index of 0.89 and a standardized root mean square residual (SRMR) of 0.054 (Supplementary Data 21). Fibromyalgia co-loaded on factor 3 with insomnia, physical activity, and traits related to pain and autoimmune response. Depression and PTSD co-loaded on factor 1 with neuroticism and generalized anxiety disorder (GAD) and on factor 6 with ADHD and suicidality. Factor 6 had a strong genetic correlation (r_g_ = 0.75) with factor 3. Suicidality co-loaded on factor 2 with substance dependence traits (Fig. 5).

### Drug Repurposing

After correction for FDR, we found no significantly enriched gene-drug pathways.

### Blood Type Phenotype

We find no differences between blood type distribution in fibromyalgia subjects compared to the general population (Supplementary Data 22).

## Discussion

Fibromyalgia is a common syndrome that impairs the well-being of about 2.7% of the population (point prevalence). Existing knowledge regarding its etiology and potential treatments is sparse^2^, especially considering its population-level impact. In this study, we identified ten independent GWS risk loci associated with fibromyalgia in EUR, one in AFR, and twelve cross-ancestry. In an MTAG analysis that leveraged pain summary statistics, we found 45 independent loci associated with fibromyalgia. These findings, alongside genetic correlation, MR and gSEM analyses, provide a genotypic basis that supports the strong observed phenotypic associations between fibromyalgia and chronic pain, mental illnesses, autoimmune diseases, and unhealthy lifestyle as reflected by physical activity. Genetic correlation analyses revealed that although fibromyalgia is more prevalent in females (75% of the cases are females)^2^, its genetic architecture in both sexes is similar. Among the genetic variants that we identified, several map to genes that are associated with a variety of neuronal pathways, pathologies, protein expression, and mental states, that can help explain the complex genetics behind the complex syndrome that is fibromyalgia:

*FBLN7* (fibulin 7; GWS in the EUR and cross-ancestry meta-analyses) has a role in inflammation^27^ and in anti-angiogenic activity^28^. It is also strongly associated with levels of MER proto-oncogene tyrosine kinase (MERTK)^29^, a regulator of immune response^30–32^ that is involved in retinal diseases^33^, in myelin phagocytosis in multiple-sclerosis (MS) patients^34^, and in systemic lupus erythematosus (SLE)^35^. The former is an autoimmune disease that was previously genetically associated with fibromyalgia through a polygenic risk score (PRS)-based pheWAS^8^, in line with a moderate genetic correlation (r_g_=0.31) we found between fibromyalgia and autoimmune diseases. The neuromodulatory role of MERTK has also been implicated in neurodegenerative diseases such as PD and AD^36^.

*PBX3*, GWS in the EUR meta-analysis and in the MTAG, is involved in various processes, from embryonic neurodevelopment to adult locomotion and respiration^37,38^. It has a role in the etiology of several types of cancer^39^ and it influences systolic BP^40,41^; it affects the structure of the brain basal ganglia^42^, which play important roles in executive functioning and emotional regulation^43^. It is also associated with gastroesophageal reflux disease (GERD)^44^, which was genetically associated with fibromyalgia through PRS^8^. In the TWAS, *PBX3* was found to be significantly enriched in the pancreas, which could indicate a potential role in the high rates of gastrointestinal disease and pancreatitis among fibromyalgia patients (4-10 times compared to the general population)^45^. In the SMR, lower enrichment of *PBX3* in the tibial artery and in non-sun-exposed skin was found to be associated with fibromyalgia too. Considering that fibromyalgia is characterized by widespread pain^1^ and that genome-wide analyses associate *PBX3* with various types of pain^46,47^, it is likely that this gene, which had a significant effect in the GWAS, MTAG, and post-GWAS analyses, is a contributor to a variety of fibromyalgia symptoms.

In three of the GWAS we conducted - the EUR, the female-specific EUR and the cross-ancestry meta-analyses, there were three different lead variants on chromosome 3, which map to three different genes but may represent the same GWS signal. This assumption is based on FUMA outputs, which pointed to a single genomic risk locus at this region in each of the analyses (despite that all three are within a ~ 100 kb region), and on LD of r^2^ > 0.6 between all three SNPs, as calculated within EUR subjects in AoU. Regional Manhattan plots of this locus visually demonstrate the proximity of these genes to each other and the distribution of significant variants across all three of them (Supplementary Figs. 13, 24). These genes are *TRAIP*, *IP6K1* and *CAMKV*, and they all have significant associations in the EUR and cross-ancestry gene-based analyses (MAGMA) too. *TRAIP* encodes the TRAF-interacting protein and is important for DNA repair^48^. It is a strong determinant of the expression of TXNDC12, a protein involved in ferroptosis^49,50^, a cell death process triggered by lipid peroxidation, which may cause neuropathic pain^51^ and increased fibromyalgia symptoms^52^. *TRAIP* is also genetically associated with ADHD^53^, cognitive functioning^54^, intelligence^55^, insomnia^56^ and pain intensity^46^ – all are traits that we found to be genetically correlated with fibromyalgia, directly (ADHD, insomnia) or indirectly (e.g., academic degree is a proxy to intelligence, chronic pain and migraine are associated with pain intensity). *IP6K1* is associated with insomnia^56^, cognitive ability^57^, and with levels of MST1^29^, a protein that affects inflammation and autoimmune response^58^. *MST1*, as well as *MST1R* (which encodes the MST1 receptor), are closely located to *TRAIP* and *IP6K1* on chromosome 3, and both had a significant effect in the gene-based analysis. *RNF123*, which resides only 83 bp from *MST1*, adds another strong association with fibromyalgia in the EUR and cross-ancestral gene-based analyses; this gene is implicated in neuroimmune pathways^59^ and was previously associated with chronic widespread musculoskeletal pain, a common symptom of fibromyalgia^10^. *CAMKV*, significant in our cross-ancestry meta-analysis, was previously shown to affect intelligence^60^, educational attainment^61,62^ and the pleiotropy between body mass index (BMI) and osteoarthritis^63^. *TRAIP, IP6K1* and *CAMKV* were all significant in the largest GWAS of PTSD published to date – the only mutual associations between that study and ours^64^.

*TSBP1-AS1* – GWS in the EUR and cross-ancestry analyses – has a strong effect on hypothyroidism and rheumatoid arthritis^44,65^, autoimmune disorders that share symptoms with fibromyalgia, like pain and muscle tenderness, in line with the genetic correlation between fibromyalgia and autoimmune diseases. *TSBP1-AS1* also has a robust effect on the levels of the immunomodulatory C2, C4, and LILRB4^29,66,67^, the deficiency of which is associated with obesity and type 2 diabetes (T2D)^68^. Both of these traits were moderately genetically correlated with fibromyalgia and may lead to lupus-like effects^69^, further establishing the role of autoimmune response in fibromyalgia. In addition, obesity, BMI, and autoimmune response are locally genetically correlated with fibromyalgia in loci that do not overlap but are adjacent ( < 1.5 MB) to *TSBP1-AS1*. Also within this region, we found BMI to be locally genetically correlated with fibromyalgia in a locus that includes *SNRPC* - significant in the EUR gene-based analysis, and previously associated with lupus erythematosus^70^ and with smoking initiation^71^. *BAG6*, closely located to *TSBP1-AS1* on chromosome 6 and significant in our GWAS, is associated with the autoimmune condition psoriasis^72^, which may cause joint pain and arthritis.

We conducted an MTAG analysis, leveraging pain summary statistics to create a better-powered genome-wide analysis of fibromyalgia. High r_g_ between the GWAS and MTAG, and almost identical genetic correlations with other traits, suggest that the MTAG results indeed represent the genetics of fibromyalgia. The effects of all the lead SNPs in the MTAG were in the same direction as in the EUR GWAS; out of 28 MTAG GWS loci in gene-coding regions, 15 had a significant effect in at least one of the EUR or cross-ancestry GWAS or MAGMA analyses, further confirming the MTAG validity. *PCLO*, GWS in the MTAG and in the female EUR GWAS, had the strongest effect in the EUR gene-based analysis. It encodes a presynaptic cytoskeletal protein involved in neurotransmitter release^73^, and implicated in neuropsychiatric disorders such as MDD, bipolar disorder, and other brain-related phenotypes^56,74,75^, consistent with pathways influencing central pain processing and the affective components of chronic pain. It also affects bone mineral density (BMD)^76^, which is associated with fibromyalgia symptoms (i.e., BMD is lower in fibromyalgia subjects)^77^, as is *DCC*^76^, which had the strongest effect in the MTAG. *DCC* encodes a protein that has an important role in axon guidance in spinal neurons^78,79^, and it is associated with various types of pain^22,80^; it is also associated with smoking initiation^71^, educational attainment^81^, and neuroticism^82^. *MAML3* and *FOXP2* were also previously associated with pain intensity^46^. Another MTAG-GWS gene of interest is *ABO*. A blood type (ABO blood group)-determining protein, *ABO* has a major impact on a variety of blood protein expression phenotypes^66,83^. Several studies point to an interaction between blood type and pain perception^84,85^. However, we did not find any phenotypic differences in blood type distribution among fibromyalgia patients compared to the general population.

Several other genes that were GWS in our GWAS and/or MTAG will be discussed briefly: *PRR16* and *UHRF1BP1* were GWS in a prior multi-ancestral GWAS of chronic pain^46^. *UHRF1BP1* and *ARHGAP15* were associated with BMD^76^. *ARHGAP15* was also found to be the strongest genetic factor contributing to diverticular disease^86^ and was associated with educational attainment, as were *KCNG2*, *CNNM2*, *EXD3* and *AFF3*^81^. *AFF3* also affects rheumatoid arthritis^87^, and is part of a list of genes that affect substance use traits such as smoking initiation and alcohol consumption, along with *SNAP91*, *CNNM2*, *NPC1* and *RABGAP1L*^71^. Though not mapping to a coding gene, rs1993709 was associated with BMI, obesity and weight^88,89^ and also with neuroticism^90^. Data obtained from GTEx portal (gtexportal.org)^91^ indicated that rs1993709 is a very strong negative eQTL of the pseudogene *RPL31P12* transcription in the cerebellum (normalized effect size = −0.87; *p* = 2.7 x 10^−16^). Though its function is not clear, *RPL31P12* had a significant effect in studies of BMI, weight and obesity^22^. The non-coding rs2587363 was previously associated with pain^9^. An intronic variant of *POLR1C* – a gene that is associated with Treacher Collins Syndrome, sensorineural hearing loss^22^ and systolic BP^44^ – POLR1C*rs186798404 was the only SNP that had a significant effect in the AFR meta-analysis, though it may be a false positive (there are no significant or close-to-significant high LD SNPs). The only significant effect in the AFR gene-based analysis was for *TEX22*, a testis-expressed gene likely associated with male fertility^92^, but also with various blood counts, both in EUR and AFR populations^44^.

Genetic correlation analysis revealed moderate-to-strong positive associations between fibromyalgia and several psychiatric traits, namely depression, suicidality, ADHD and PTSD. These correlations are in line with high comorbidity between fibromyalgia and these traits^11–13^. In people with both PTSD and fibromyalgia, the onset of fibromyalgia symptoms is usually later than the occurrence of the traumatic event^93^, a time course consistent with possible causality. While MR revealed bidirectional causality between these traits, the causal effect of PTSD (as exposure) on fibromyalgia (as outcome) was much stronger (although not for the sub-phenotypes of PTSD), suggesting that the genetic liability to PTSD may be more likely to cause fibromyalgia than vice versa. The same is true regarding depression and suicidality, but not ADHD: MR results show that genetic liability to fibromyalgia is more likely to cause ADHD; it is possible that what is reflected here are concentration and attention problems (including what is sometimes referred to as “brain fog”) – known to be common in fibromyalgia – that might be coded as ADHD. The moderate r_g_ between fibromyalgia and neuroticism aligns with a known genetic correlation between neuroticism and various pain-related traits^94–96^, and with dominant neuroticism in fibromyalgia patients compared to other personality traits of the Big-Five model, as was found in a personality-trait questionnaire conducted among fibromyalgia patients^97^.

The psychiatric traits described above are strongly associated with chronic pain^95,96^, one of the most dominant symptoms of fibromyalgia. Chronic pain was the trait most strongly genetically correlated with fibromyalgia in our study (r_g_ = 0.80 ± 0.02), alongside a relatively high correlation with migraine - a more specific pain-related trait. Migraine was also the trait most strongly affected by fibromyalgia in the MR analysis, though this effect is potentially influenced by horizontal pleiotropy. In a gSEM analysis, fibromyalgia co-loaded with chronic pain and migraine, as well as with endometriosis, autoimmune response, insomnia, and physical activity, but not with psychiatric traits. This suggests that fibromyalgia may be contextualized more as a pain and autoimmune trait than a psychiatric trait, even though the genetic correlations with psychiatric traits such as PTSD and depression are quite high. Genetic correlations between fibromyalgia and opioid use disorder (OUD; r_g_ = 0.41 ± 0.05) and CanUD (r_g_ = 0.33 ± 0.03) are of importance here, due to the use of opioids to alleviate pain, and the growing use of cannabis in recent years to treat symptoms of fibromyalgia^2,16,98,99^. We found associations between fibromyalgia and two genes previously associated with OUD^100^, representing a complex set of effects related to both OUD and fibromyalgia: *FOXP2*, previously associated with pain^46^, but also with a range of psychiatric and neurological traits such as externalizing behavior^101^, ADHD^102^, and insomnia^56^, had a significant effect in our fibromyalgia MTAG; and *NICN1*, associated with MST1 protein levels^29^, intelligence^57^, educational attainment^103^, and metabolic syndrome^104^, had an effect on fibromyalgia in the EUR gene-based analysis. We found no mutual GWS associations between fibromyalgia and CanUD. MR analysis revealed that fibromyalgia has a weak (yet statistically significant) causal effect on CanUD and OUD risk. The effect of OUD (as exposure) on fibromyalgia (as outcome) was stronger than any other effect in this MR analysis, indicating that the genetic variants associated with OUD risk may also be causal with respect to fibromyalgia. This is consistent with findings regarding increased pain sensitivity due to chronic opioid use^105^. The moderate genetic risk of developing dependence of opioids and cannabis among fibromyalgia patients should be taken into account when prescribing them as treatment.

A moderate negative genetic correlation between fibromyalgia and physical activity (r_g_ = −0.5 ± 0.03) reinforces the importance of physical activity as a possible treatment for fibromyalgia symptoms^2^ (alternatively, that fibromyalgia itself causes reduction in physical activity, as seen in the bidirectional results in the MR analysis). Along with a positive genetic correlation we found between fibromyalgia and BMI, obesity, high BP and T2D, our observations provide additional support for a biological connection between a healthy lifestyle and fibromyalgia. The moderate r_g_ fibromyalgia had with endometriosis is also backed by the strong phenotypic association between these traits^106^. Negative genetic correlations between fibromyalgia and executive functioning and with academic degree indicate a genetic relationship underlying the phenotypic association between fibromyalgia and impaired cognitive performance^107^. The moderate genetic correlation we identified between fibromyalgia and autoimmune diseases (r_g_ = 0.31 ± 0.03), along with the autoimmune roles of several GWS genes in our study, point to the possibility that fibromyalgia has autoimmune components. This notion was suggested previously^108^, with studies linking fibromyalgia and autoimmune diseases through genetic and inflammatory pathways^8,109^. Certain autoimmune diseases such as SLE are more prevalent in people of AFR ancestry^110,111^. While data regarding the prevalence of fibromyalgia in AFR is sparse, the prevalence of fibromyalgia in our AFR meta-analysis was twice as high as for EUR, suggesting that fibromyalgia may potentially be more prevalent in subjects of AFR ancestry. Conceivably this could relate to the effects of autoimmune disorders. Although there are known phenotypic associations between fibromyalgia and neurodegenerative diseases such as AD and PD^112,113^, genetic correlation analysis revealed no significant association between these traits.

LAVA analysis revealed local genetic correlations between fibromyalgia and several traits in regions that map to genes of interest. Four traits – executive functioning (negative r_g_ value), CanUD, neuroticism and PTSD (positive r_g_) – were correlated with fibromyalgia in a region on chromosome 11 that maps to *TTC12*, which had a significant effect in the EUR gene-based analysis. This gene affects neuroticism^114^, alcohol dependence^115^, and nicotine dependence traits^116,117^, and is an important factor in the expression of NCAM1, a cell adhesion molecule that is genetically associated with a variety of substance use traits^71,116–118^. It also is physically adjacent to *DRD2*, which encodes the dopamine D2 receptor and is associated with psychiatric and behavioral traits such as MDD^119^, anxiety^120^ neuroticism^82^, and a variety of substance use traits^71,121,122^*. NRXN1*, which maps to a region that genetically correlates fibromyalgia with academic degree, was previously associated with educational attainment^81^. This gene, which had a significant effect in the EUR gene-based analysis, encodes a protein (neurexin-1) that participates in neurotransmission and is expressed in synaptic regions^37^. *GRIA1* had a significant effect in the EUR gene-based analysis. It encodes the GluA1 subunit of the AMPA receptor, a major mediator of synaptic excitatory transmission; it is associated with insomnia^56^ and personality disorders^22^, and it also maps to a region that genetically correlates fibromyalgia with PTSD. *NRXN1* and *GRIA1* are two of several genes that were identified in this study known to influence brain function and morphology; amongst the other such genes identified herein are *PBX3*^42^, *PCLO*^73^, *MAPRE3*^123,124^, and *DPYSL5*^123,124^. This is in line with the high enrichment of pain-related genes in the brain that was found in a recent trans-ancestral GWAS of pain intensity^46^. Specifically, *GRIA1*-mediated alterations in AMPA receptors may influence the function of GABAergic interneurons^125,126^ and alter pain sensation^127^, consistent with the high enrichment of pain-related genes in GABAergic neurons demonstrated in the same previous study^46^. Administration of the cannabis-derivatives Δ9-tetrahydrocannabinol (THC) and cannabidiol (CBD) induced alterations in *Gria1* expression in rodents^128–130^, consistent with growing evidence of cannabinoid-glutamatergic associations^131^, suggesting a potential pathway through which cannabis may ameliorate fibromyalgia symptoms^98,99^.

Nine genes identified in the gene-based analysis map to a region on chromosome 2 that is locally positively genetically correlated with fibromyalgia and depression (there is a possible signal at the same region, which can be seen in Fig. 1a, though there are no GWS variants). Four of these genes (*DPYSL5*, *MAPRE3*, *AGBL5*, *CGREF1*) are associated with blood triglyceride levels^29,132,133^, two (*CENPA*, *SLC35F6*) with BMD^76^, and two (*MAPRE3*, *DPYSL5*) with brain morphology^123,124^. Another gene, *KHK*, encodes an enzyme with a major role in fructose metabolism.

This study included fibromyalgia data from four large biobanks. In AoU, UKBB and Finngen, heritability estimates were between 11–19%, and females were 82–92% of the total number of cases. In MVP, however, heritability was considerably lower (3%), and females accounted for only 19% of the fibromyalgia cases (compared to 7% of the sample). Nevertheless, the incidence of fibromyalgia in MVP was much higher, compared to the other cohorts we investigated (Table 1). While the different case distribution among sexes in MVP is attributable to the sex imbalance in the cohort, the low heritability is puzzling and may relate to the military nature of MVP. Indeed, soldiers tend to be more physically active in their daily routine compared to civilian population^134^. As MVP subjects are veterans, there may be higher likelihood that they were exposed to environmental factors that contributed to fibromyalgia symptoms during their service (e.g., injuries, traumatizing events). These two factors may explain the high prevalence but low genetic heritability of fibromyalgia among MVP participants. Nevertheless, genetic correlations between the EUR fibromyalgia cohorts were high (r_g_ values ranged between 0.72 and 0.95; Supplementary Data 8), indicating that the fibromyalgia phenotype in all the cohorts we analyzed likely represents the same trait. A strong genetic correlation (r_g_=0.73 ± 0.06), between male and female EUR participants, as well as great similarities between the genetic correlations of males’ and females’ fibromyalgia datasets with other traits (Supplementary Data 9; Fig. 2), indicates that fibromyalgia likely represents the same trait in both sexes.

Our GWAS analyses yielded a total of 15 genomic loci associated with fibromyalgia across different ancestries, ten of which map to a coding gene – and the MTAG resulted in 35 additional GWS loci, 22 of which map to a coding gene. Several of these genes are associated with traits that share with fibromyalgia symptomatic characteristics like pain, muscle tenderness, and insomnia; these may be important for the nociplastic pain experienced by fibromyalgia patients. This concept reflects central sensitization, a heightened central-nervous system response to sensory input, and is supported by genetic studies showing shared heritability across multiple pain traits, including this study. Other genes are associated with physical measures of general health like BP and BMI. Some of the genes we identified in this study are associated with neuronal pathways and with structural changes in brain regions that mediate emotional regulation and executive functioning, with mental and cognitive phenotypes such as ADHD, educational attainment and cognitive functioning, and with substance use traits. In addition, at least three of the genes we found are associated with autoimmune disorders. These findings are all in line with moderate genetic correlations observed between fibromyalgia and phenotypes of general health (physical activity, T2D, high BP, BMI, obesity) and of substance use (CanUD, PAU, OUD), and moderate-to-strong genetic correlations of fibromyalgia with chronic pain, with psychiatric traits (ADHD, depression, suicidality, PTSD), and with autoimmune traits.

Our findings may indicate that fibromyalgia is a combination of several factors that create a complex trait, which can be categorized under several different factors that contribute to its phenotypic expression, with gSEM suggesting that it may be considered mainly as pain- and autoimmune-associated.

This study has limitations. First, in two of the three cohorts in which we conducted a sex-stratified GWAS, the number of male participants with fibromyalgia (i.e., cases) was low and we did not detect GWS loci. Nevertheless, as discussed earlier, the genetic correlation between male and female fibromyalgia datasets was high, suggesting that the trait is similar or identical between the sexes. Second, only one GWS locus was detected in the AFR meta-analysis, and none in AMR, probably due to low sample sizes, but also possibly due to gaps in diagnosis and/or phenotype definition in these populations^135,136^; non-significant heritability of fibromyalgia in those populations was also likely due to low sample size. Third, there were substantially more controls than cases in all cohorts (cases accounted for 1% to 12.5% of the various samples); such imbalances are typical in studies of this kind, but could potentially bias power and stability, particularly for low-frequency variants. In our study, however, all the lead SNPs had MAF > 7%. Fourth, fibromyalgia prevalence differs between geographic regions and populations (for example: 9.3% in Tunisia, 8.8% in Turkey and 0.4% in Greece, compared to the world prevalence of 2.7%^137^) and also between different studies of the same region, suggesting that the true prevalence of this disorder in the population can be biased due to practices of diagnosis and environmental-cultural differences (in diet and exercise, as well as in symptom expression). Nevertheless, it may also represent actual genetic differences between populations that are underrepresented in large genetic biobanks, an issue that needs to be addressed in future studies, when more data regarding these populations are available. Fifth, the MTAG analysis was conducted using GWAS summary statistics of a broad pain phenotype defined by Finngen^22^ and which includes multiple ICD codes of different pain measures. This increased the power of the analysis, but at the risk of false-positive results and loss of phenotype specificity. Despite the substantial genetic correlation between the two sets of summary statistics (fibromyalgia and pain; r_g_ = 0.72), the analysis may have introduced bias; therefore we did not treat this as our primary analysis.

In total, our results provide novel insights into the genetic architecture of fibromyalgia, providing information about its genetic association with pain, autoimmune response, psychiatric traits, and lifestyle. These results allow for a deeper understanding of fibromyalgia and may provide tools for future identification of genetic risk factors that affect this complex disorder, which in turn may help with diagnosis. Genetic correlations between fibromyalgia and physical activity, as well as with traits that are highly associated with nutrition and cardiovascular function, support the importance of adopting a healthier lifestyle for the treatment and/or prevention of fibromyalgia. We studied multiple populations. While we identified the greatest number of findings in EUR, there was also independent GWS findings in AFR. All populations contributed to increasing findings in our trans-population meta-analysis. There is future potential for medication repurposing, personalization of treatments, and eventually, based on improved understanding of underlying biology, development of new treatments and even prevention strategies for fibromyalgia, but considering the lack of results for the former in the present analyses, this will require future studies with more subjects and better power.

## Methods

This research was not restricted or prohibited in the setting of any of the included researchers. All studies were approved by the relevant local institutional review boards and ethics review committees. AoU was approved by the AoU IRB, governed by the US National Institutes of Health (NIH). UKBB was approved by the North West Multi-center Research Ethics Committee as a research tissue bank. MVP was approved by the Veterans Affairs Central Institutional Review Board.

### Cohorts

We analyzed data from AoU, version 8 (whole-genome sequencing), MVP (imputation conducted using the 1000 Genomes project^138^ as a reference panel), and UKBB (imputation conducted using the Haplotype Reference Consortium^139^, in combination with the merged UK10K and 1000 Genomes reference panels^140^). For full description of genotyping and quality control procedures of AoU, MVP, and UKBB (EUR1, British EUR population; EUR2, non-British EUR population), see refs. ^20,141,142,143^, respectively. In the meta-analysis, we also included summary statistics for fibromyalgia from Finngen subjects (release 12)^22^.

### Initial analyses for phenotype definition

To include only confirmed cases of a studied disorder, a phenotype is often defined by a stringent ICD-10 code-based phenotype definition (a): ≥ 2 outpatient and/or 1 inpatient visit/s. In this study, to create well-powered analyses, we aimed to assemble a larger sample size by considering inclusion of (b) fibromyalgia subjects meeting a less-stringent ICD-10 phenotype definition (1 outpatient visit), or (c) self-report (of having been diagnosed with fibromyalgia). To do this, we conducted separate GWAS of fibromyalgia, assigning subjects as cases according to the three different phenotype definitions: stringent ICD-10, less-stringent ICD-10 (with a single outpatient diagnosis considered sufficient to define affection), and self-report. For all the analyses that involved ICD-10 codes, we used ICD-10 code M79.7. Cases distribution and overlap between ICD-10 and self-report phenotype definitions in AoU and in UKBB are presented in Supplementary Fig. 1. We then calculated the genetic correlations between GWAS based on these differing case definitions, to ascertain genetic similarity between these phenotypes. We conducted these preliminary phenotype definition tests in each of AoU, MVP and UKBB separately.

In each of the cohorts, we defined fibromyalgia two or three ways, depending on data availability: a stringent ICD-10 code definition, less-stringent ICD-10 code definition, and self-report. Subjects that fit more than one of these phenotypes were excluded from this part of the analysis completely, i.e., they were not defined as cases nor controls (subjects that fit, for example, both strict ICD-10 code and self-report, were excluded; this exclusion applied only for these analyses conducted to evaluate the genetic relationship of subjects defined by these three phenotype methods). Only subjects that fit one category but not the others were included in the analysis as cases of the relevant category. Subjects that did not fit any category were classified as controls. Any subject classified as a case in one of these analyses, was excluded from the others (i.e., there was no situation where a subject was used as a case for one analysis and as a control subject in another). We conducted GWAS of these phenotypes separately, removing one individual from each pair of related subjects (kinship coefficient cutoff = 0.1), retaining as many cases as possible. GWAS were conducted with PLINK 2.0 using logistic regression, with sex, age, and the first ten genetic principal components (PCs) as covariates.

In AoU, we extracted three different fibromyalgia phenotypes: stringent ICD-10 (n_cases_ = 2,932, n_controls_ = 212,390), less-stringent ICD-10 (n_cases_ = 1,951, n_controls_ = 210,571), and self-report (“Including yourself, who in your family has had fibromyalgia? Select all that apply. – Self”; n_cases_ = 5,339, n_controls_ = 208,813). In MVP, we extracted two different fibromyalgia phenotypes: stringent ICD-10 (n_cases_=24,041, n_controls_ = 384,196) and less-stringent ICD-10 (n_cases_=20,847, n_controls_ = 379,286). In UKBB, we extracted two different fibromyalgia phenotypes: stringent ICD-10 (EUR1: n_cases_ = 2,328, n_controls_ = 374,731; EUR2: n_cases_ = 329, n_controls_ = 52,072) and self-report (“Have you ever been told by a doctor that you have had any of the following conditions? -Fibromyalgia syndrome -Yes”; EUR1: n_cases_ = 1,666, n_controls_ = 374,840; EUR2: n_cases_ = 228, n_controls_ = 52,081; non-stringent data (defined elsewhere based on outpatient visits) were not available), and conducted an inverse variance weighing meta-analysis of EUR1 and EUR2 results of each phenotype (using METAL^144^) before further analyses. For all the analyses, we used LDSC^145^ to calculate the liability-scaled heritability estimate (h^2^) and intercept (population prevalence was set at 2.7%^2^). We also tested the genetic correlations for the fibromyalgia phenotypes between these fibromyalgia case definitions, defined by stringent ICD-10 code definition, less-stringent ICD-10 code definition, and self-report. A strong genetic correlation between these sub-cohorts would confirm that these different definitions of fibromyalgia correspond to genetically similar traits, enabling the valid inclusion of subjects of these different cohorts. We also included in our analysis EUR fibromyalgia summary statistics from Finngen, release 12^22^, defined by the stringent ICD-10 code (n_cases_= 3623, n_controls_ = 357,549).

### Main Analysis

After we confirmed that the various fibromyalgia phenotypes (stringent ICD-10, less-stringent ICD-10 and self-report) are strongly genetically correlated (see previous section) – suggesting that they are likely representing a consistent trait – we defined a singular fibromyalgia phenotype in AoU, MVP and UKBB: subjects with stringent ICD-10 (M79.7), less-stringent ICD-10 and self-report definitions of fibromyalgia, including overlapping samples, were defined as cases. All other subjects were defined as controls. In AoU and MVP we conducted separate GWAS in subjects of European (EUR), African (AFR) and Latin American (AMR) genetically-defined ancestries. In UKBB we conducted the analysis in EUR. In each cohort, after removing one individual from each pair of related subjects (kinship coefficient cutoff = 0.1), retaining as many cases as possible, GWAS was conducted with PLINK 2.0 using logistic regression, with sex, age and the first ten genetic PCs as covariates. Variants with minor allele frequency (MAF) < 0.1%, Hardy-Weinberg equilibrium (HWE) *p* < 1x10^−6^, or imputation quality < 0.6 were excluded. In UKBB, we meta-analyzed EUR1 and EUR2 results before further analyses. For each ancestry in each cohort we also conducted sex-stratified GWAS. EUR, AFR, AMR and cross-ancestry SE-based meta-analyses (for all subjects and sex-stratified) were conducted using METAL^144^, including heterogeneity analysis. The non-sex-stratified EUR meta-analysis included fibromyalgia summary statistics from the Finngen biobank^22^. In all the analyses we applied a standard genome-wide multiple testing correction (*p* < 5×10^−8^). The results were visualized in a Manhattan plot using the R package qqman^146^. Regional Manhattan plots were created using FUMA^147^. Linkage disequilibrium (LD) was calculated between three variants on chromosome 3 (rs2681780, rs71080556, and rs763622663) that were identified as lead SNPs in three different GWAS. These variants are located within different genes yet in proximity to each other, and may represent the same GWS signal. This analysis was conducted using PLINK2 in EUR subjects in AoU.

### FUMA and MAGMA gene-based and gene set analyses

We used FUMA^147^ to define genomic risk loci and map the lead variants’ exact location in the genome, pointing to a specific gene when applicable. We used MAGMA^148^, implemented in the FUMA platform, to conduct a gene-based analysis of the EUR, AFR, AMR and cross-ancestry meta-analyses. In the gene-based analyses, input SNPs were mapped to 19,069 protein-coding genes in EUR, 10,995 in AFR, 18,856 in AMR and 19,173 cross-ancestry, and corrected for false-discovery rate (FDR).

### Genetic Correlations and SNP-based heritability

We used LD score regression (LDSC)^145^ based on the linkage disequilibrium reference from the 1000 Genomes data^138^ for all EUR cohorts. We calculated the liability-scaled SNP-based heritability estimate (h^2^) and intercept of fibromyalgia in every cohort separately (population prevalence was set on 2.7%^2^), and the genetic correlations (r_g_) between fibromyalgia phenotypes in the different cohorts. After meta-analysis, we calculated h^2^ for the meta-analyzed fibromyalgia trait and its r_g_ with a list of traits related to psychiatric disorders, autoimmune diseases, sleep, neurodegeneration, and general health^22,44,56,64,95,119,121,143,149–157^, phenotypically associated with fibromyalgia according to prior literature^1,2,112,113^. We also conducted similar analyses for the sex-stratified meta-analyses of fibromyalgia in EUR and for the EUR MTAG, using the same summary statistics for the comparative traits that we used for the main analysis (we did not use sex-specific summary statistics of other traits due to low availability of well-powered GWAS of most of the traits we analyzed, and because we aimed to maintain the analyses with as few differences as possible, for better comparison between the results). After Bonferroni correction for 108 tests (4 fibromyalgia phenotypes x 27 traits), the statistical significance threshold was set at *p* = 0.0005.

### Multi-Trait Analysis of GWAS (MTAG)

To enhance the statistical power of the EUR fibromyalgia GWAS, we used MTAG^158^, a method that enables joint analysis of traits that are genetically correlated with each other, and provides estimates of trait-specific effects. We used pain, a similar phenotype to the trait with the strongest genetic correlation with fibromyalgia we found using LDSC (chronic pain). A diagnosis of fibromyalgia may be categorized as pain, which raised the risk of using overlapping samples diagnosed under overlapping traits. Although MTAG is appropriate for overlapping samples, to minimize confounds we avoided using datasets with potentially high case overlap with fibromyalgia, and therefore we used summary statistics for pain among Finngen subjects, with 237,944 cases and 261,418 controls^22^. This pain phenotype was defined by 16 different diagnoses associated with limb, back, neck, head, and abdominal pain. There were only 817 fibromyalgia subjects in the same group, < 0.2% of the number of the pain patients, illustrating the low susceptibility for case overlap. The r_g_ between these two traits was 0.72 ( ± 0.026). For an additional, more inclusive analysis, we aimed to capture the wider complexity of fibromyalgia as both a pain- and psychiatric-related trait. For this analysis we used depression for the MTAG analysis, because of the availability of a large-scaled GWAS of EUR major depressive disorder (MDD) that does not include any of the cohorts we used in our study. We used MDD summary statistics from a large meta-analysis^24^, taking a leave-one-out (LOO) approach, excluding subjects from MVP, UKBB, Finngen – and also 23andMe due to use restrictions (these four cohorts were part of the set of cohorts included in the aforementioned MDD study). After exclusion, the MDD dataset included 181,654 MDD cases and 862,012 controls. The r_g_ between fibromyalgia and MDD was 0.63 ( ± 0.025), and between pain and MDD was 0.56 ( ± 0.021). In both sets of analysis, MTAG was conducted using fibromyalgia as the main trait, with variants restricted to only those common to the two GWAS, with MAF > 0.01. Because MDD had a relatively lower genetic correlation with fibromyalgia and with pain than the recommended threshold for MTAG (rg ≥ 0.7), we focused on the MTAG of fibromyalgia leveraged by pain (fibromyalgia MTAG leveraged by pain and MDD together is reported in the supplementary material).

### Mendelian Randomization (MR)

We conducted MR analyses to estimate the causality between fibromyalgia and 25 traits that had significant effects in the genetic correlations analysis (see previous section), assessing fibromyalgia both as exposure and outcome. All MR analyses were conducted using MRlap^159^, which is appropriate for MR analysis with potentially overlapping cohorts. We ran the inverse variance weighted (IVW) model, with two p-value thresholds of 1 × 10^−5^ and 1 × 10^−8^ to select genetic instruments. Due to a low number of variants left after pruning using the stricter threshold of 1 x 10^−8^, we report only the 1 × 10^−5^ threshold analysis in our results (MR analysis using a p-value threshold of 1 x 10^−8^ is reported in the supplementary material). Instrument pruning was conducted based on an LD threshold of 0.05. For every analysis, MRlap performs a correction for overlapping samples and other potential biases such as outliers and presents the statistical difference between the observed and corrected values. Where the difference between these values was significant (*p* < 0.05) we presented the corrected values in the results section; where the difference was not significant, we presented the observed values. To account for potential pleiotropy and for violations of instrumental variable assumptions, we applied complementary two-sample MR methods^25^, including MR-Egger, weighted median, inverse-variance weighted (IVW), simple mode, and weighted mode. PTSD could not be included in the two-sample MR analyses because the available summary statistics do not provide effect size and standard error^64^, which are essential for these analyses. After Bonferroni correction for 340 tests (MRlap: 25 traits as exposure and outcome, in two different p-value thresholds; two-sample MR: 24 traits as exposure and outcome, measured in five different tests), the p-value significance threshold set at 1.47 x 10^−4^. Pleiotropy was tested for all significant causal effects using the MR-Egger intercept test (p-value threshold after Bonferroni correction for 48 tests set at 0.001).

### Local Analysis of covariant Association (LAVA)

We used LAVA^160^ to calculate local genetic correlations between fibromyalgia and 27 traits of interest in EUR. The genome was divided into 2,495 genomic regions to provide minimal LD between the regions and maintain an approximately equal size of the regions of ~1 MB. Breakpoints between regions were computed according to the LD between neighboring SNPs as described previously^160^, maintaining regions as relatively independent. Univariate local correlations were calculated for each trait, and only regions that reached significance (*p* = 2 x 10^−5^ after Bonferroni correction) were used to calculate genetic correlations among the cohorts (9295 regions in 27 pairs). Bonferroni correction for 9295 tests yielded a statistical significance threshold of *p* = 5.38 x 10^−6^.

### Cross-ancestry genetic correlations

We applied Popcorn^26^ to calculate the cross-ancestry genetic correlations between fibromyalgia in AFR and AMR and fibromyalgia in EUR, as well as other traits in EUR.

### Transcriptome-Wide Association Study (TWAS)

We conducted TWAS using GTEx_v8^161^, which provides expression data of 49 tissues in EUR samples. We used the 1000 Genomes dataset as LD reference. Using FUSION^162^, we identified associated genes, then processed the results to distinguish conditionally independent genes. In 49 tissues, there were a total of 300,187 genes measured (an average of 6126 ± 2787 per tissue). We therefore used a Bonferroni correction for 300,187 tests (0.05/300,187) to set a *p*-value threshold of 1.66 x 10^−7^.

### Summary-based Mendelian Randomization (SMR)

We conducted SMR to test whether genetic variants that regulate gene expression (eQTLs) influence fibromyalgia risk. We conducted this analysis across thousands of genes in 51 tissues (an average of 3,239 genes per tissue), using data from GTEx_v8^161^, implemented in the SMR portal^163^. GTEx_v8 data presents the influence of various SNPs on gene expression across different tissues. In this analysis, gene expression served as the exposure, and summary statistics of our fibromyalgia EUR meta-analysis as the outcome; for each tissue, we tested whether gene expression had an inferred causal effect on fibromyalgia (i.e., we tested whether variants that regulate gene expression affect fibromyalgia). Heterogeneity in dependent instruments (HEIDI) test was conducted to assess whether the association was consistent with a shared genetic signal rather than distinct variants in LD. Across all genes and tissues included in the analysis, we conducted 165,231 tests. After Bonferroni correction, p-value threshold was set 3.03 x 10^−7^.

### Genomic Structural Equation Modeling (gSEM)

We conducted a gSEM analysis^164^ on fibromyalgia and 22 traits that had significant genetic correlations with fibromyalgia. Three of these traits – academic degree, executive functioning and physical activity, which had a negative genetic correlation with fibromyalgia – were reverse-coded before the analysis. Exploratory factor analysis (EFA) was performed followed by confirmatory factor analysis (CFA) to identify and confirm the factor structure. EFA models containing 1–9 factors were evaluated based upon eigenvalues, sum of squared (SS) loadings, cumulative variance explained, and the distribution of variance explained across the respective factors. CFA models were assessed using traditional fit indices^164^.

### Drug repurposing

We used the drug.MATADOR database, which is implemented in ShinyGo 0.82^165^, to identify potential drug-target interactions, using 173 genes that had a significant effect in at least one of the methods we used in this study – GWAS, MAGMA, MTAG and TWAS – in any of the ancestries we studied.

### Blood type phenotype

After we found a significant effect of *ABO* (a blood-type determining gene) in the MTAG, and based on known associations between blood type and pain perception^84,85^, we compared the ABO blood group blood type phenotype between fibromyalgia subjects and the general population, using data available in UKBB.

### Software

This study used publicly available software, as follows: PLINK 2.0: plink-ng/2.0 at master · chrchang/plink-ng · GitHub LDSC^145^: https://github.com/bulik/ldsc. METAL^144^: https://github.com/statgen/METAL. MRlap^159^: https://github.com/n-mounier/MRlap. MAGMA^148^: https://cncr.nl/research/magma/. LAVA^166^: https://github.com/josefin-werme/LAVA. MTAG^158^: https://github.com/JonJala/mtag. gSEM^164^: https://github.com/GenomicSEM/GenomicSEM. FUSION^162^: https://github.com/gusevlab/fusion_twas. MR-MEGA^167^: https://github.com/NIH-CARD/MA_MA_meta. SMR^163^: https://github.com/jianyanglab/SMR-Portal.

### Reporting summary

Further information on research design is available in the Nature Portfolio Reporting Summary linked to this article.

## Supplementary information

Supplementary Information

Description of Additional Supplementary Files

Supplementary Data

Reporting Summary

Transparent Peer Review file

## Source data

Source Data

## Data Availability

Summary statistics (by population, and meta-analysis) generated in this study have been deposited in the figshare database and will be made available also through the Gelernter Lab website. The MVP data used in this study are available in the GenHub VA Repository for genome-wide study results [https://genhub.va.gov/public/sumstats]. AoU data are available in the AoU database [https://www.researchallofus.org]. UKBB data are available in the UKBB database [https://www.ukbiobank.ac.uk]. Finngen data are available in the Finngen data release 12. GTEx data are available in the GTEx portal [https://www.gtexportal.org]. The drug.MATADOR data are available in the ShinyGo 0.82 portal [https://bioinformatics.sdstate.edu/go82]. All other data supporting the findings of this study are available in the article and its Supplementary Information files. Source data are provided with this paper.
