# Supplementary material for: The genetics of fibromyalgia and its relationships to psychiatric and medical traits
Source: Nat Commun. 2026 Jul 28;17:6248. doi: 10.1038/s41467-026-75256-6 (PMC13415558; doi:10.1038/s41467-026-75256-6)
Supplement: Supplementary file 1 — Supplementary Information [file 41467_2026_75256_MOESM1_ESM.pdf]

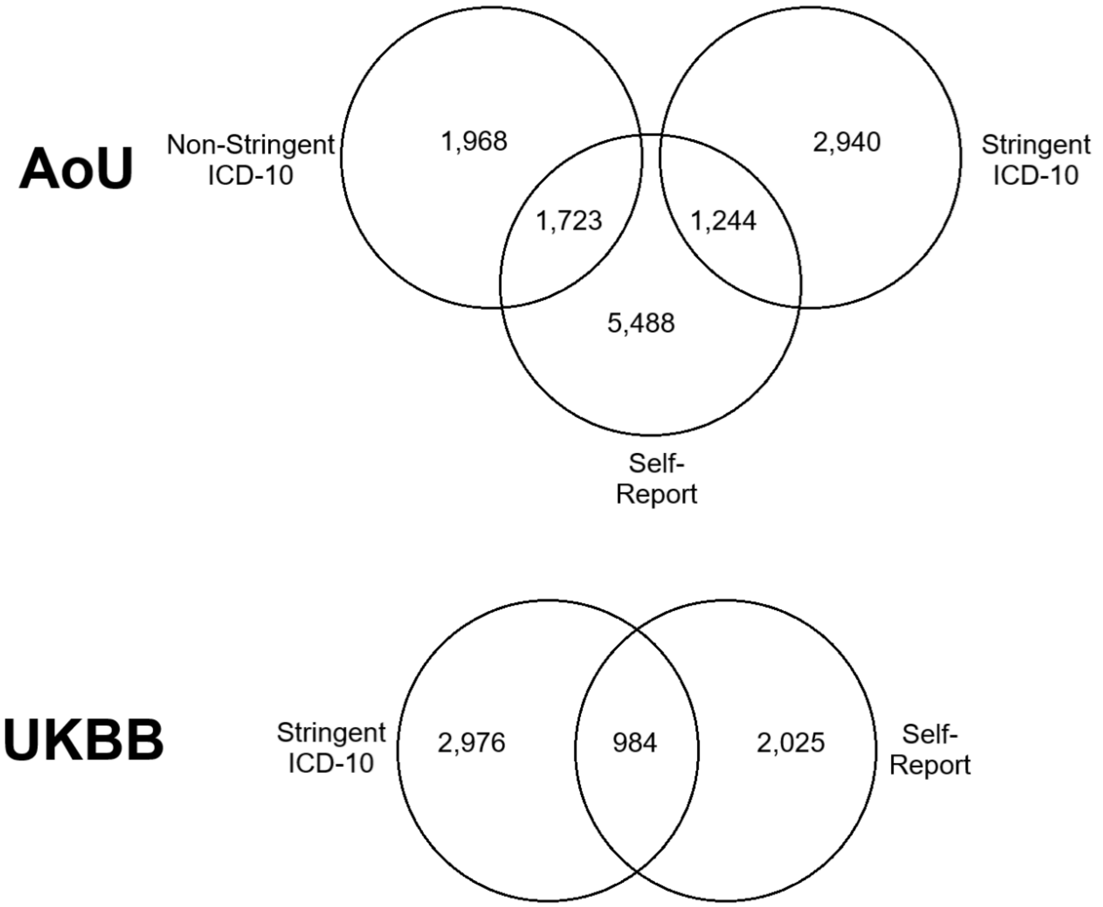

Supplementary Figure 1: **Fibromyalgia Case Distribution.** Cases distribution by phenotype definition (ICD-10 / self-report) in fibromyalgia subjects in All of Us and in UKBB. The figures are pre-kinship removal, therefore the figures are slightly different from those that were used for GWAS.

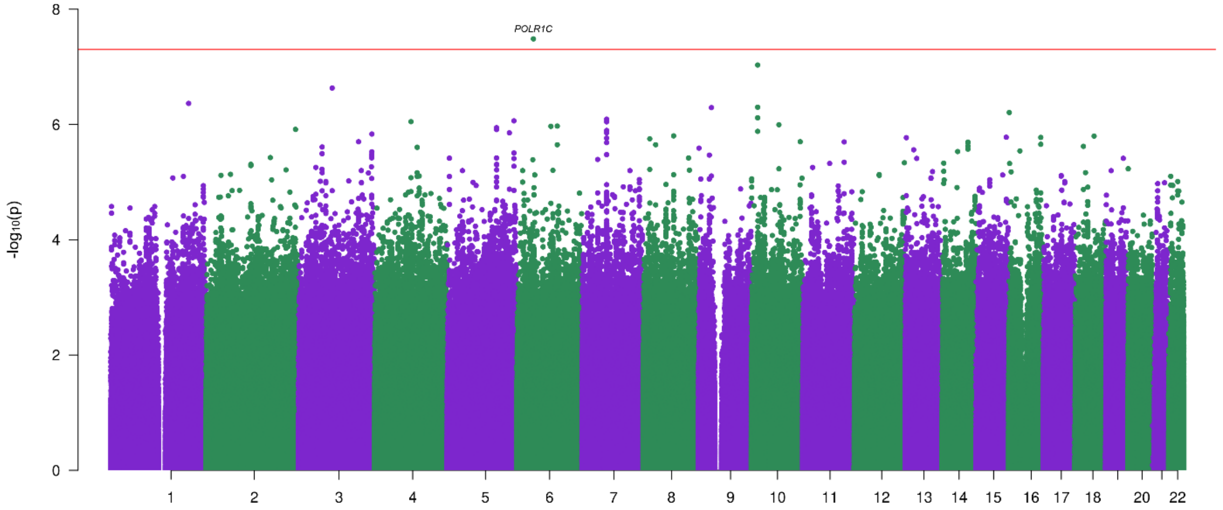

Supplementary Figure 2: **Fibromyalgia GWAS in AFR.** GWAS meta-analysis of fibromyalgia in AFR population (n_{total}=180,959, n_{eff}=57,560).

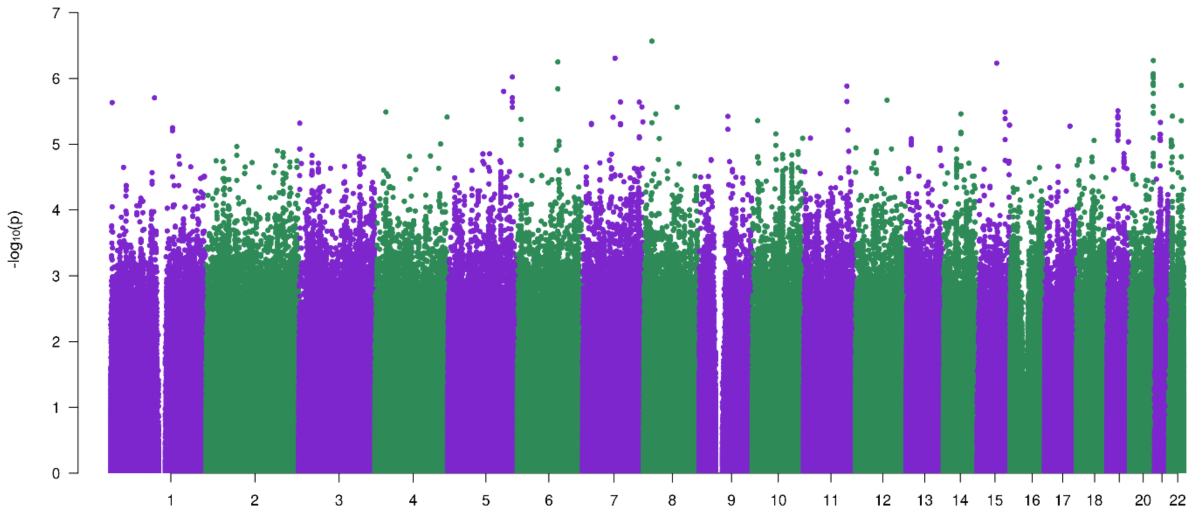

Supplementary Figure 3: **Fibromyalgia GWAS in AMR.** GWAS meta-analysis of fibromyalgia in AMR population (n_{total}=106,089, n_{eff}=24,645).

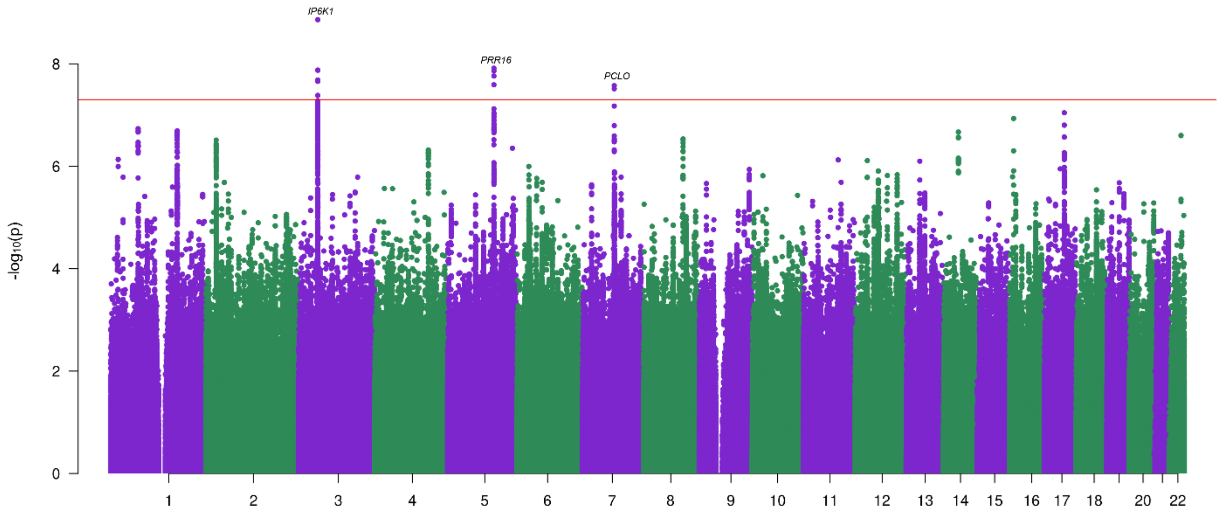

Supplementary Figure 4: **Fibromyalgia GWAS in EUR females.** GWAS meta-analysis of fibromyalgia in EUR population - females (n_{total}=401,602, n_{eff}=87,471).

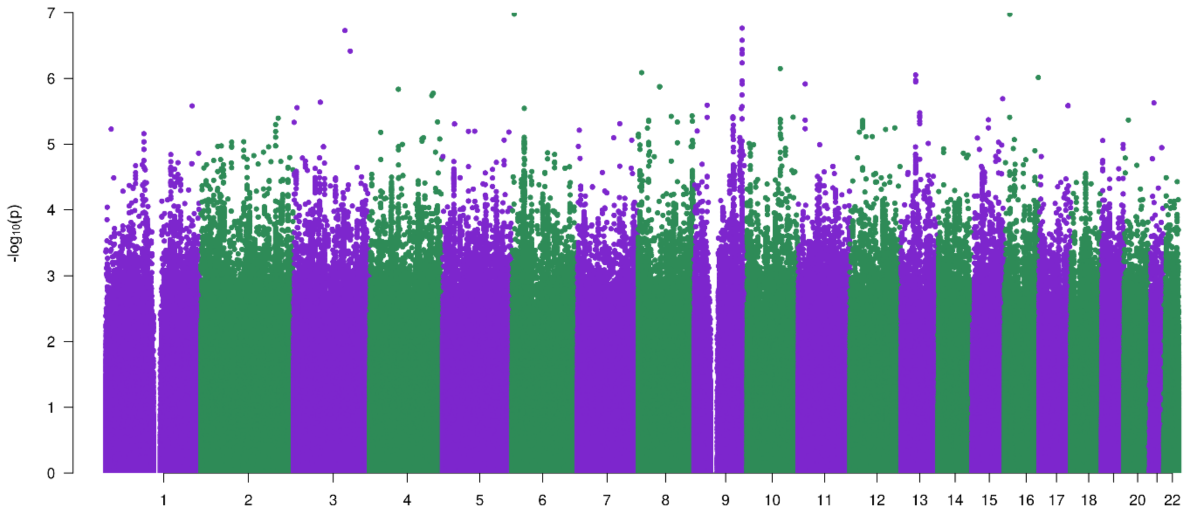

Supplementary Figure 5: **Fibromyalgia GWAS in EUR males.** GWAS meta-analysis of fibromyalgia in EUR population - males (n_{total}=689,978, n_{eff}=135,670).

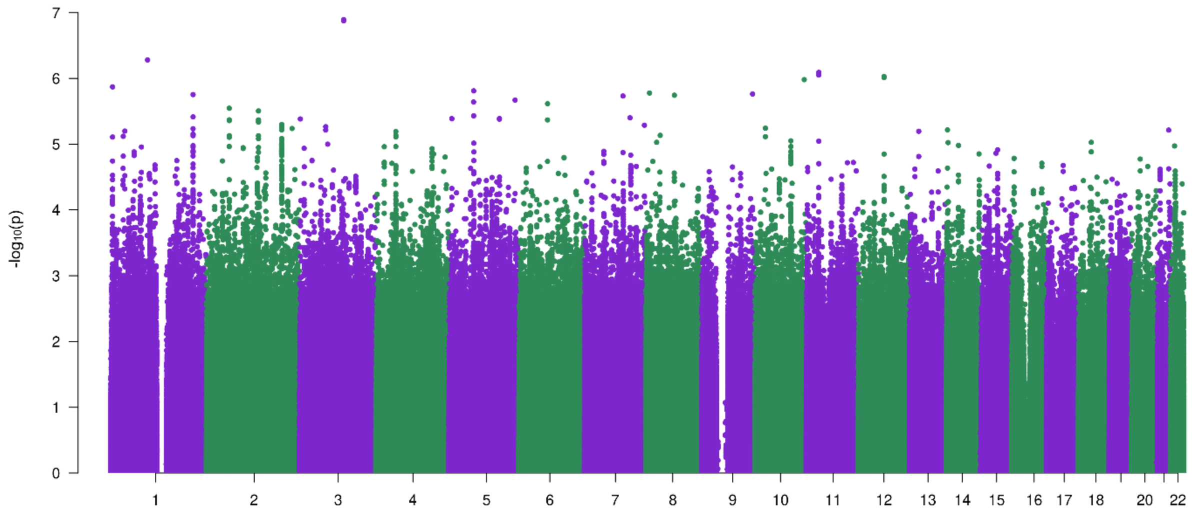

Supplementary Figure 6: **Fibromyalgia GWAS in AFR females.** GWAS meta-analysis of fibromyalgia in AFR population - females (n_{total}=57,078, n_{eff}=20,376).

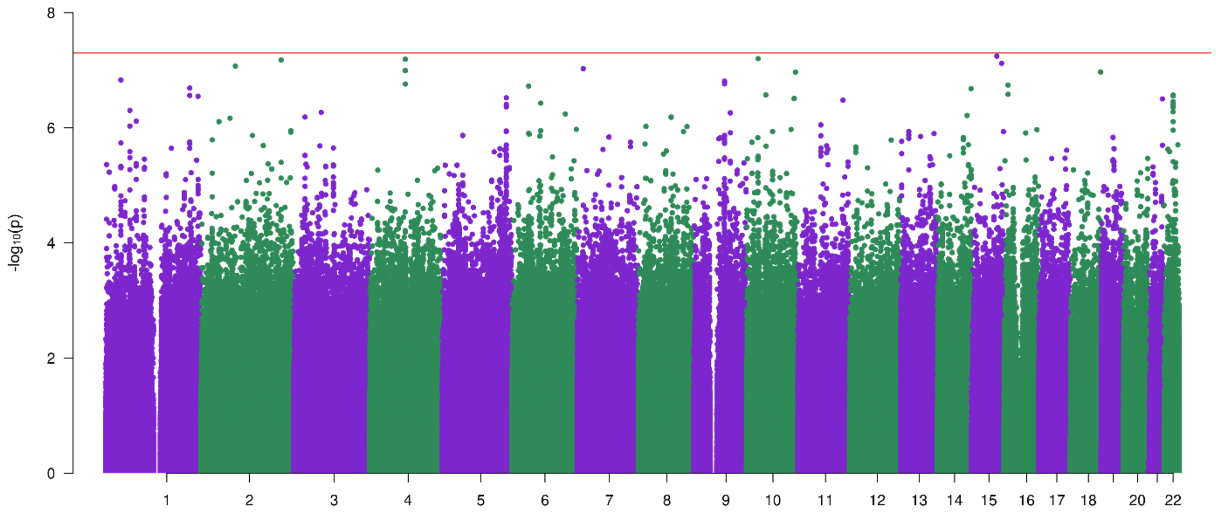

Supplementary Figure 7: **Fibromyalgia GWAS in AFR males.** GWAS meta-analysis of fibromyalgia in AFR population - males (n_{total}=123,969, n_{eff}=37,145).

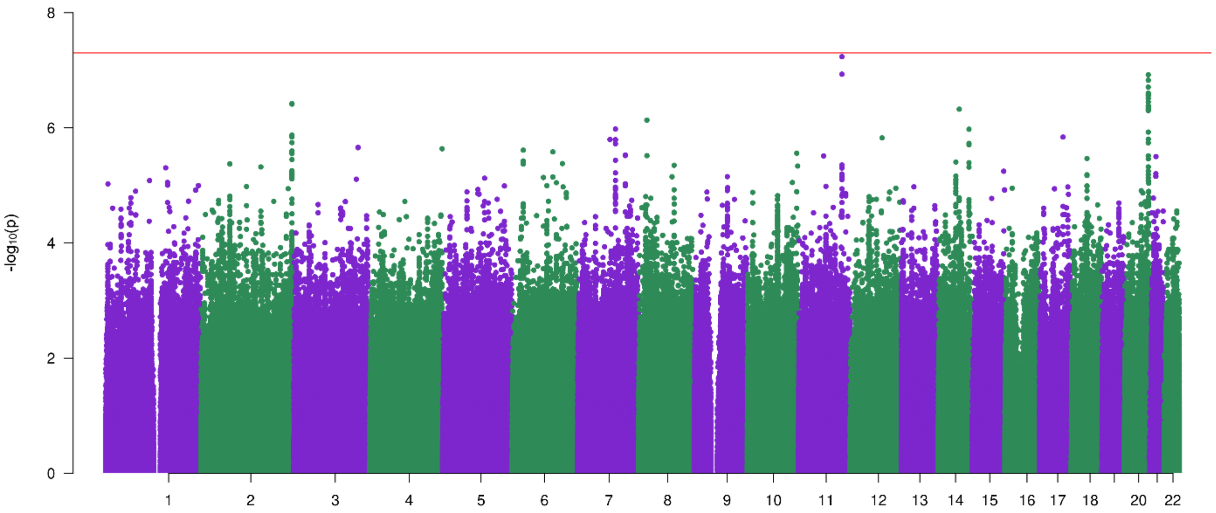

Supplementary Figure 8: **Fibromyalgia GWAS in AMR females.** GWAS meta-analysis of fibromyalgia in AMR population - females (n_{total}=48,890, n_{eff}=11,889).

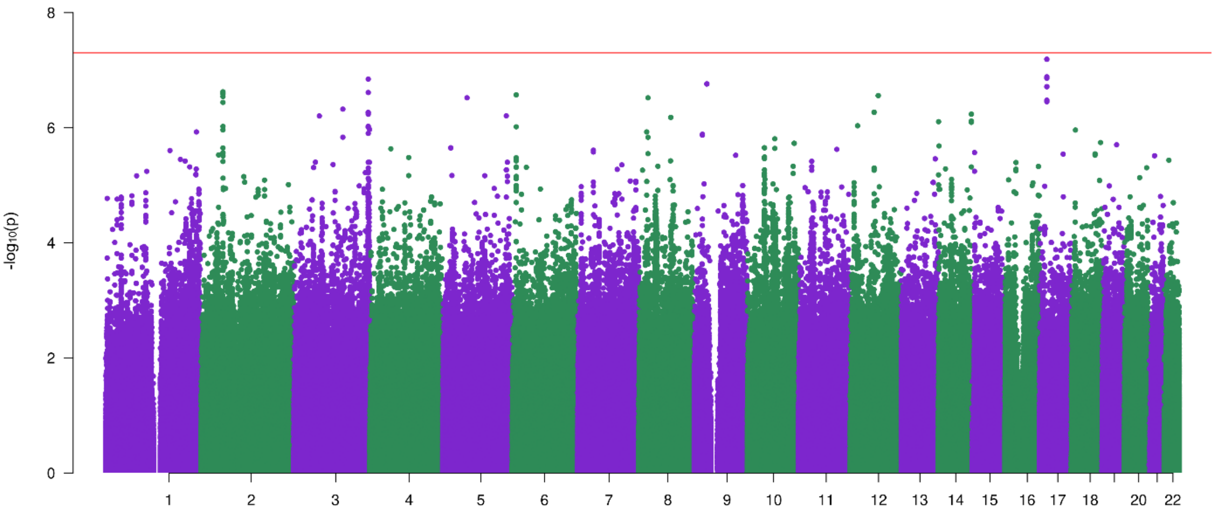

Supplementary Figure 9: Fibromyalgia GWAS in AMR males. GWAS meta-analysis of fibromyalgia in AMR population - males (n_{total}=57,270, n_{eff}=12,761).

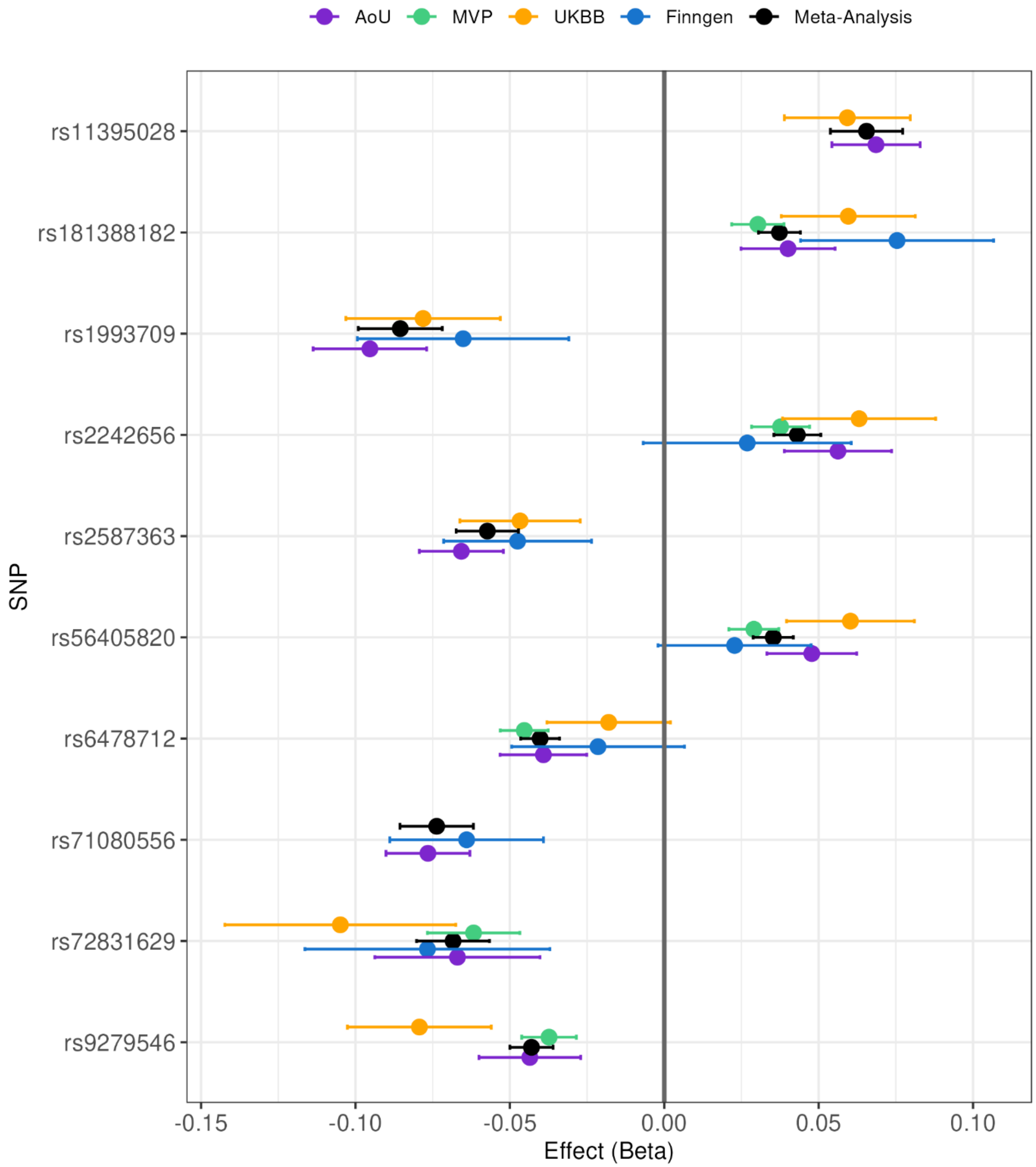

Supplementary Figure 10: **Fibromyalgia EUR Meta-analysis – effects by cohort.** significant variants effect sizes across cohorts that were included in the EUR meta-analysis of fibromyalgia. Error bars represent standard error [AoU: All of Us; MVP: Million Veteran Program; UKBB: UK Biobank].

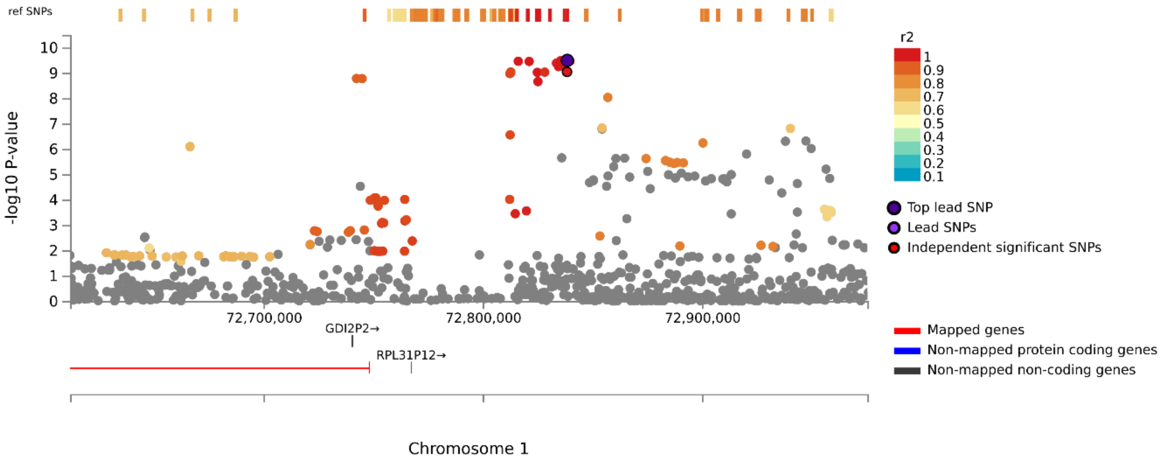

Supplementary Figure 11: **Regional Manhattan Plot.** rs1993709 (EUR meta-analysis).

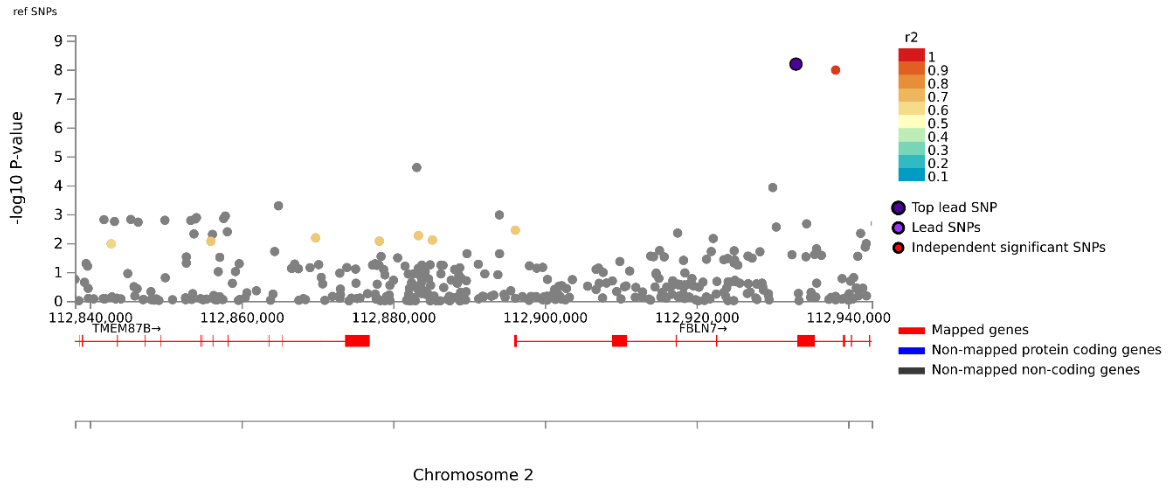

Supplementary Figure 12: **Regional Manhattan Plot.** rs72831629 (EUR meta-analysis).

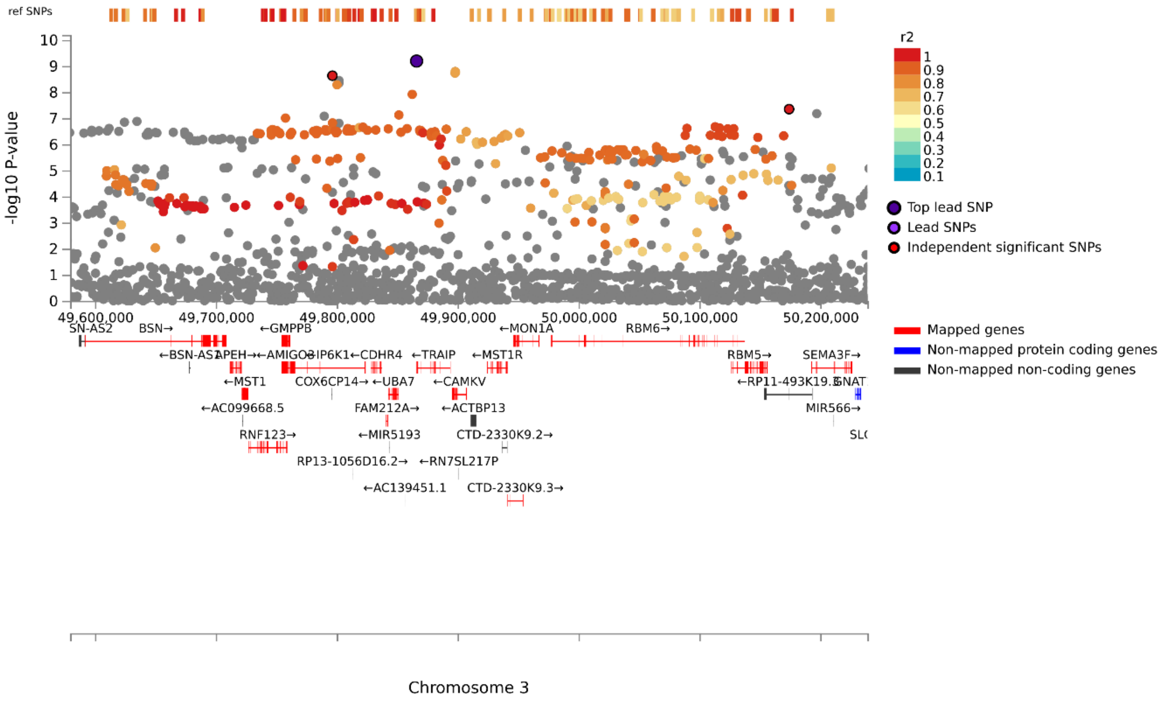

Supplementary Figure 13: **Regional Manhattan Plot.** rs71080556 (EUR meta-analysis).

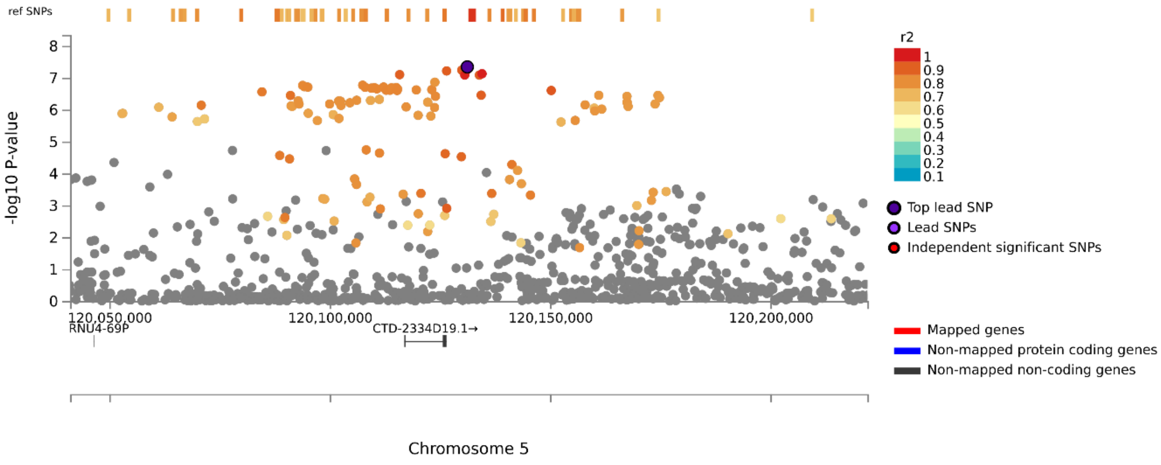

Supplementary Figure 14: **Regional Manhattan Plot.** rs56405820 (EUR meta-analysis).

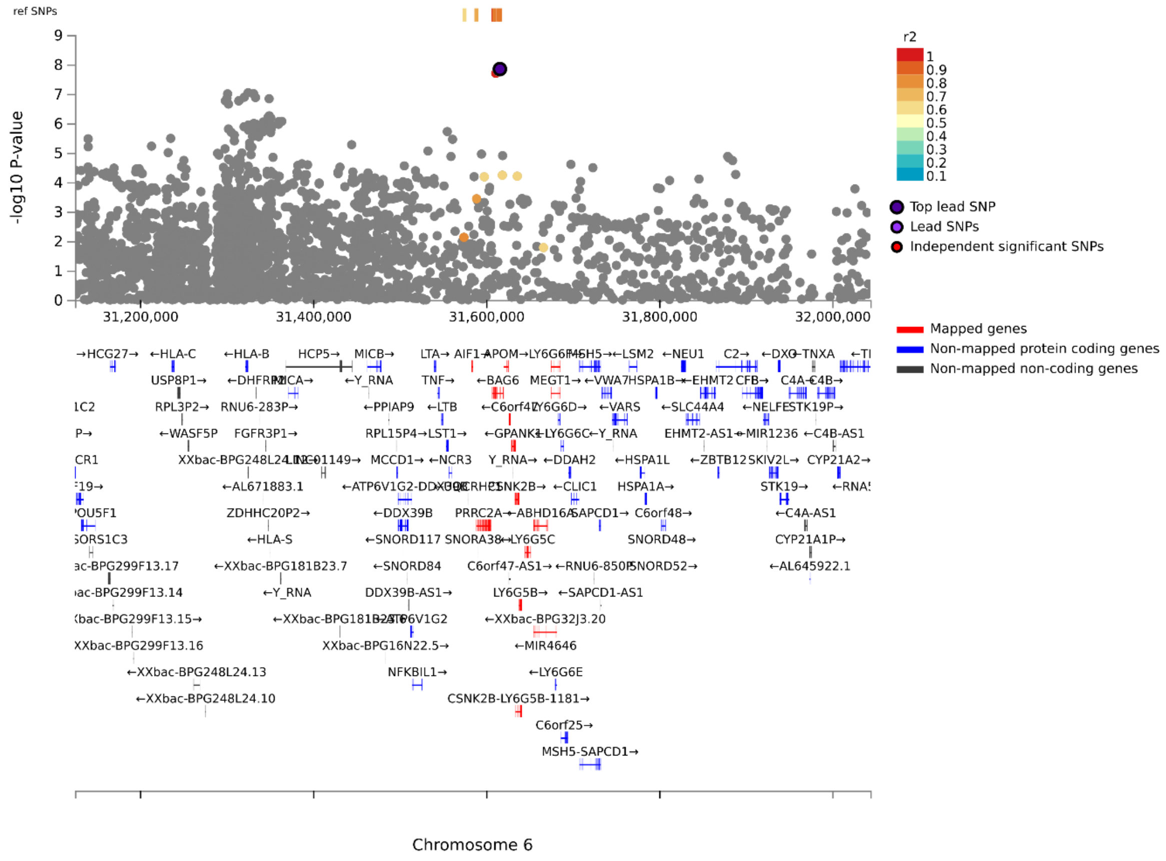

Supplementary Figure 15: **Regional Manhattan Plot.** rs2242656 (EUR meta-analysis).

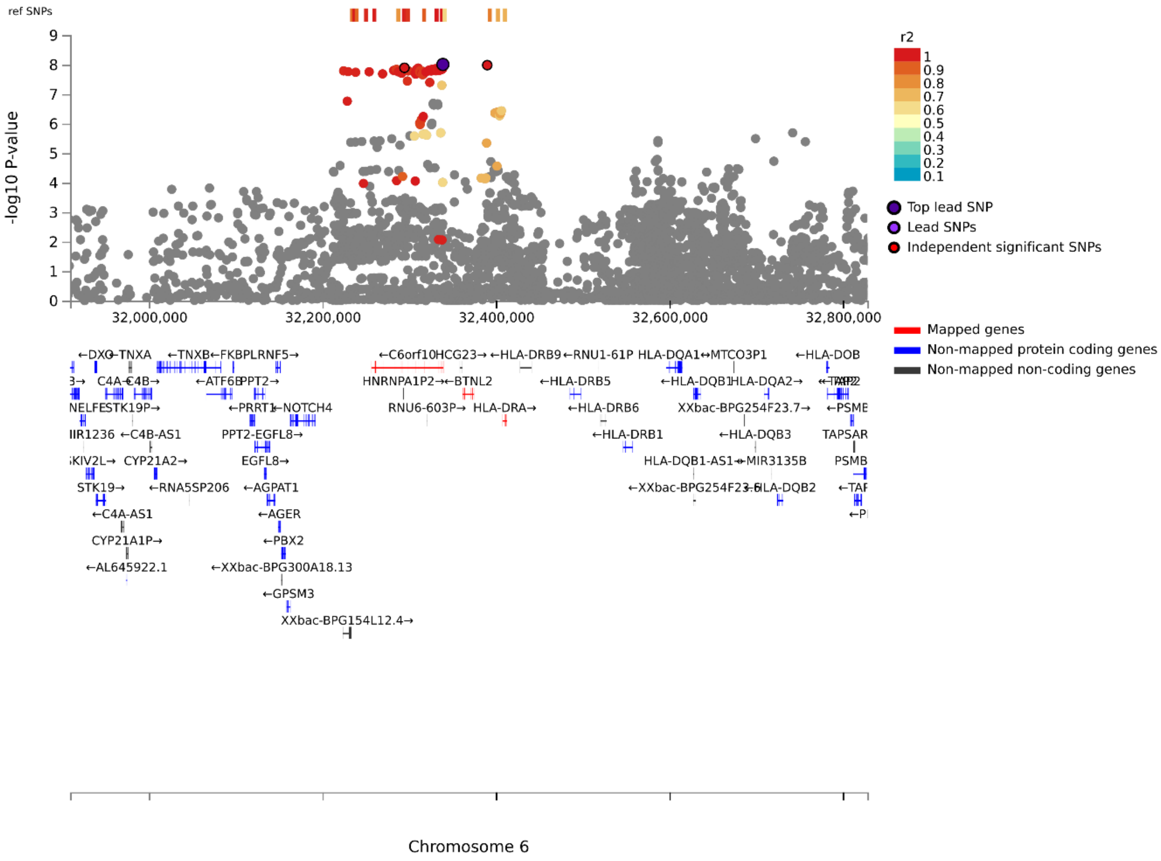

Supplementary Figure 16: Regional Manhattan Plot. rs9279546 (EUR meta-analysis) [note: *TSBP1* is also known as C6orf10].

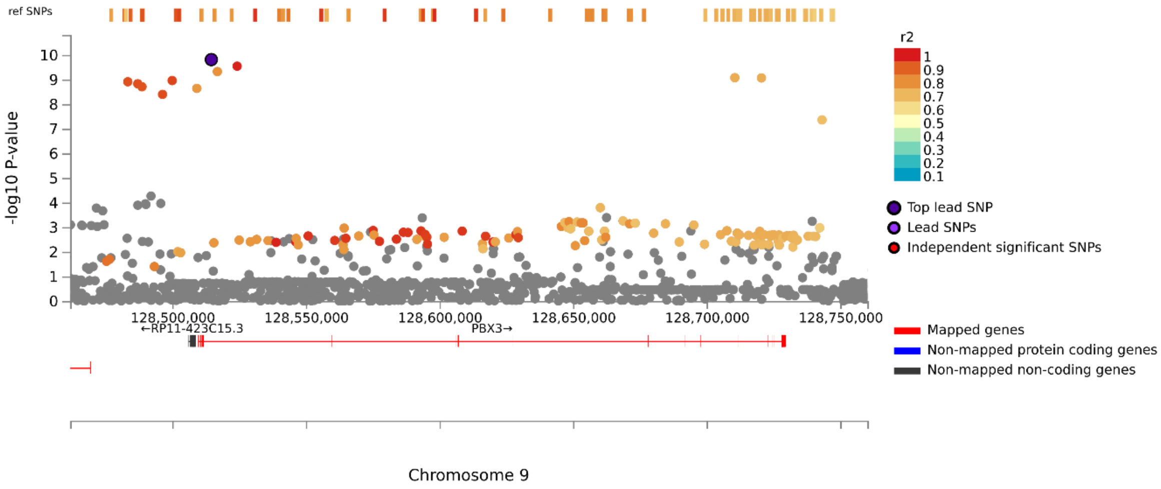

Supplementary Figure 17: **Regional Manhattan Plot.** rs6478712 (EUR meta-analysis).

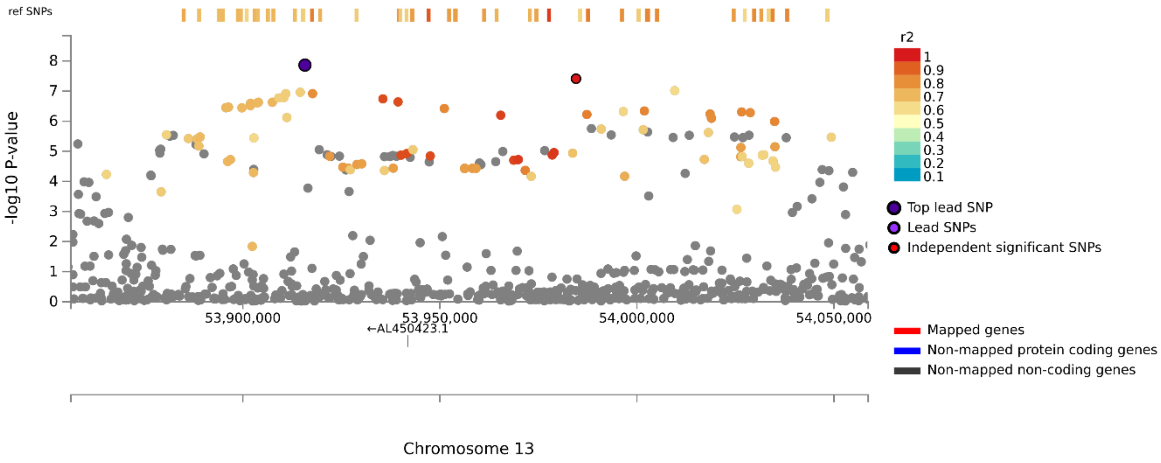

Supplementary Figure 18: **Regional Manhattan Plot.** rs2587363 (EUR meta-analysis).

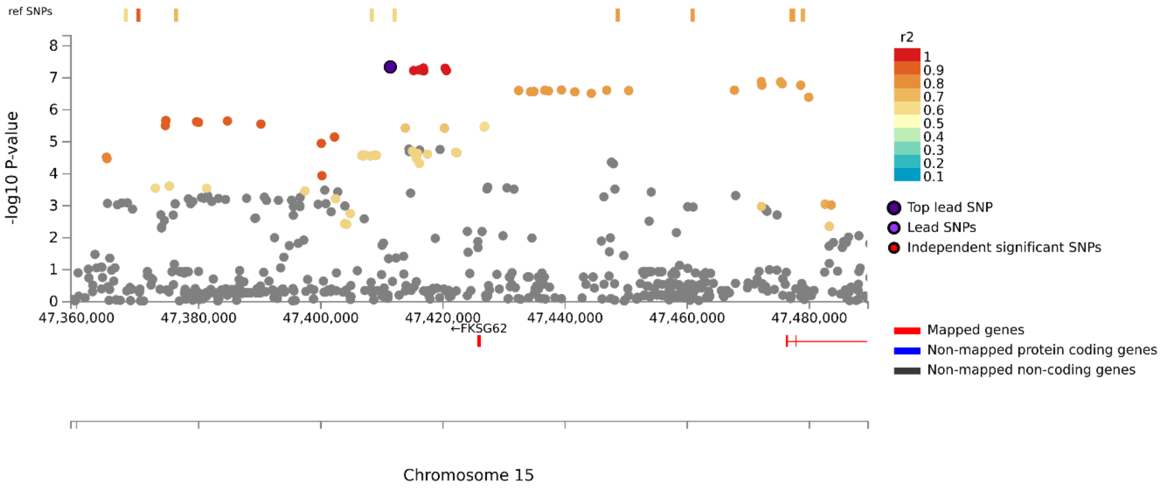

Supplementary Figure 19: **Regional Manhattan Plot.** rs181388182 (EUR meta-analysis).

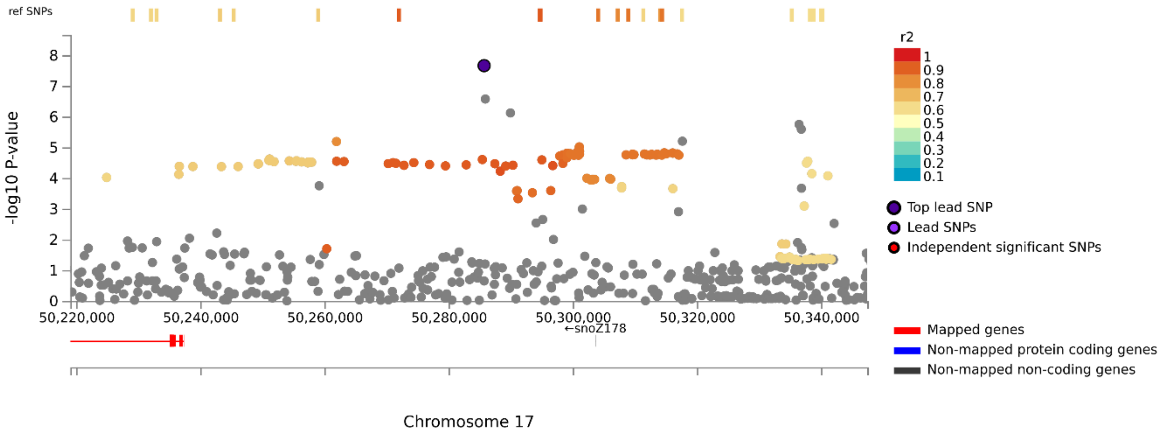

Supplementary Figure 20: Regional Manhattan Plot. rs11395028 (EUR meta-analysis).

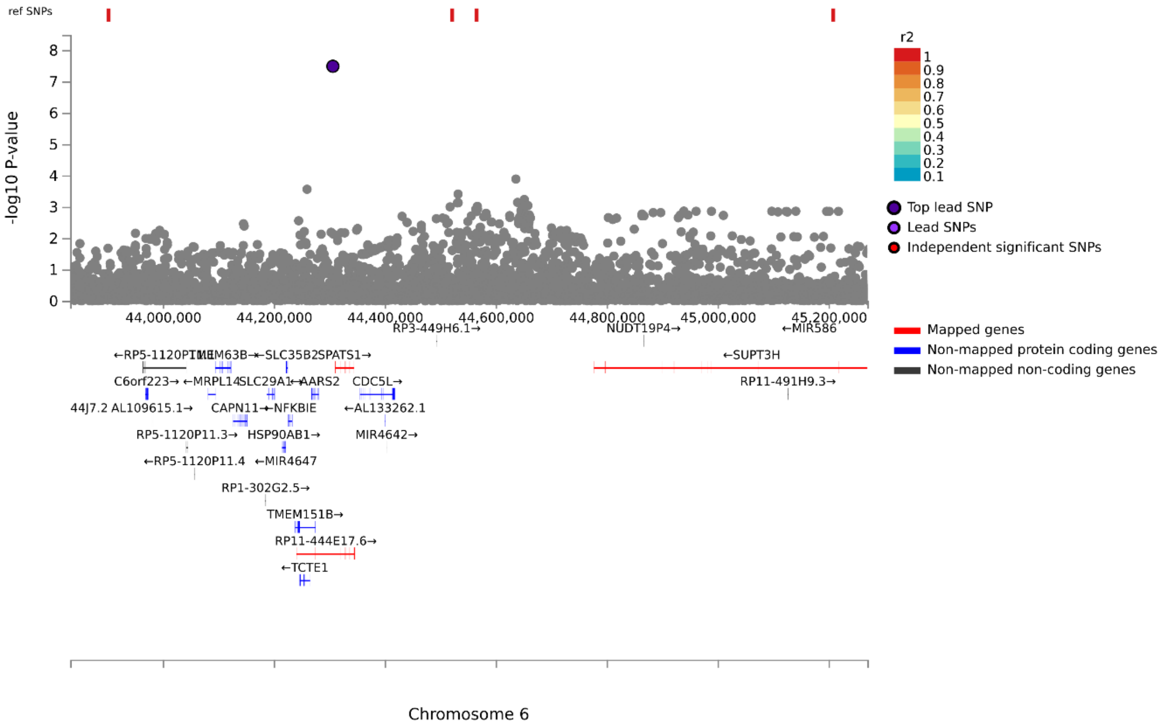

Supplementary Figure 21: Regional Manhattan Plot. rs186798404 (AFR meta-analysis).

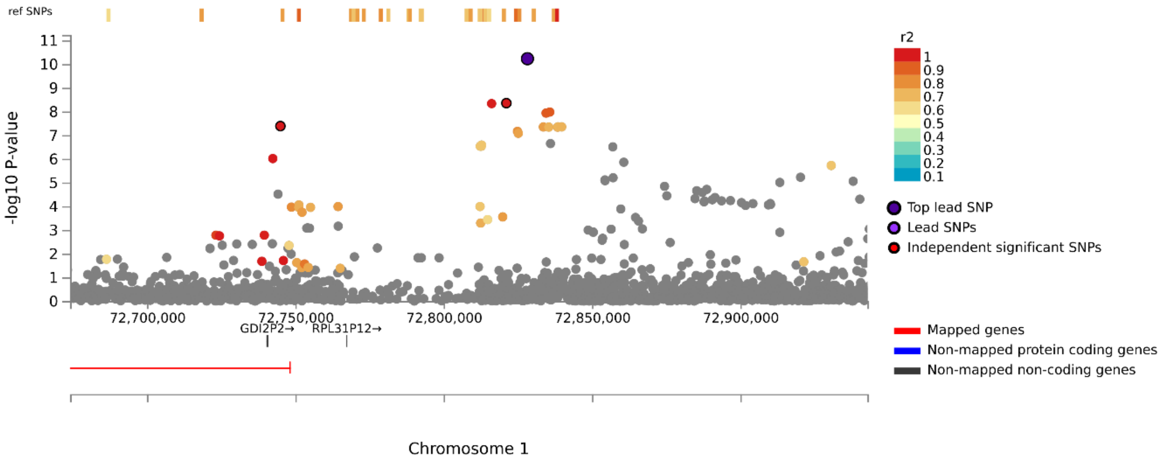

Supplementary Figure 22: Regional Manhattan Plot. rs10889947 (cross-ancestry meta-analysis).

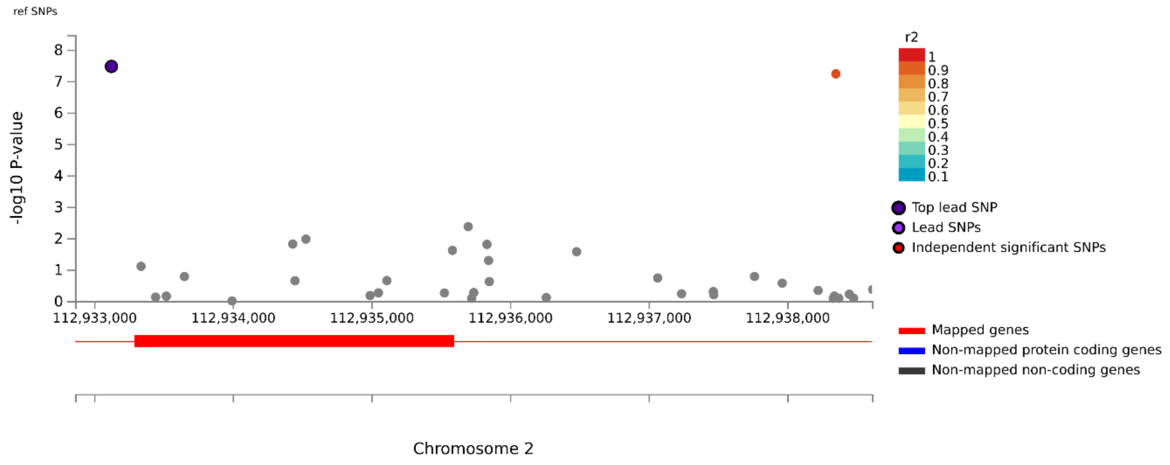

Supplementary Figure 23: Regional Manhattan Plot. rs72831629 (cross-ancestry meta-analysis).

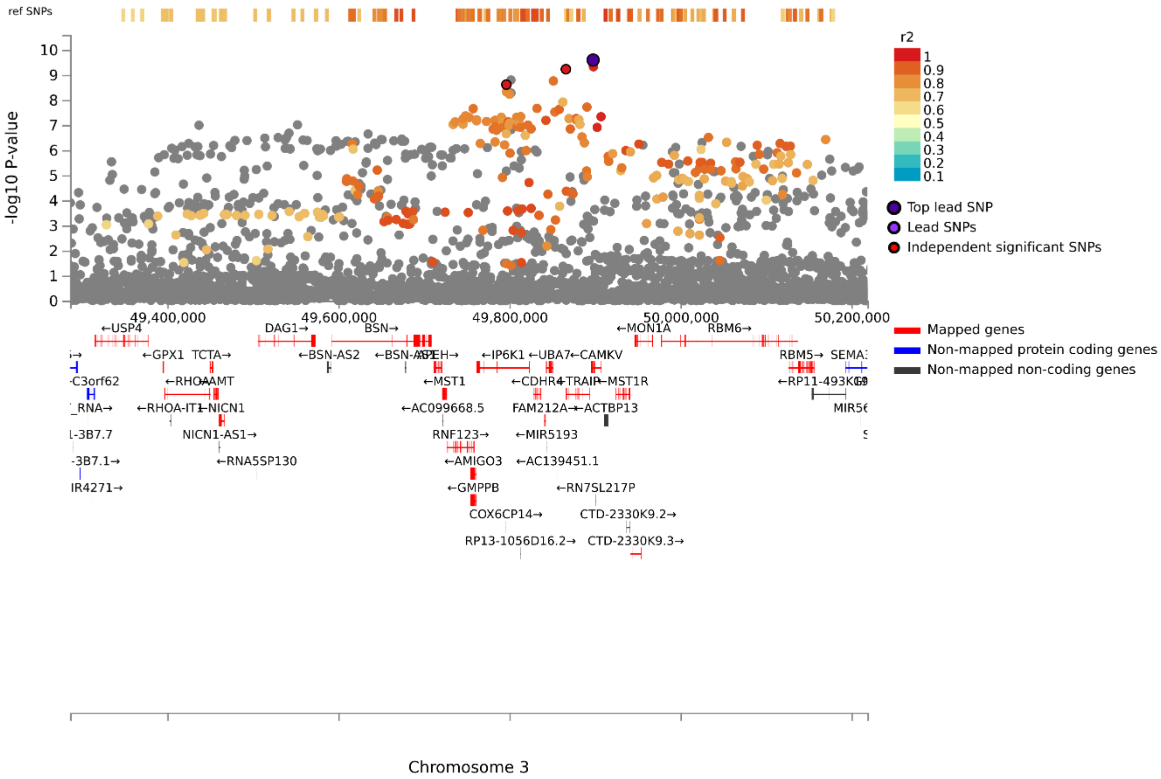

Supplementary Figure 24: **Regional Manhattan Plot.** rs2681780 (cross-ancestry meta-analysis).

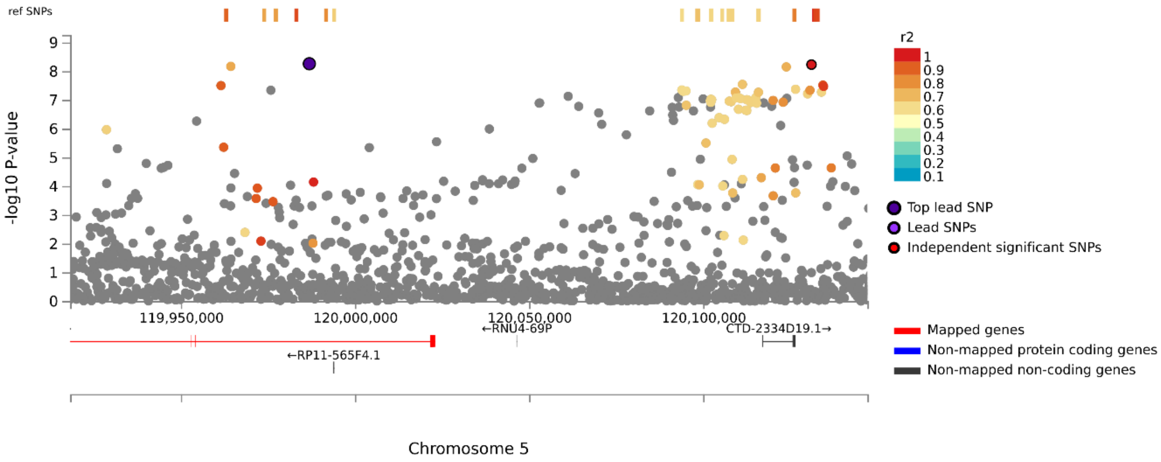

Supplementary Figure 25: **Regional Manhattan Plot.** rs190161089 (cross-ancestry meta-analysis).

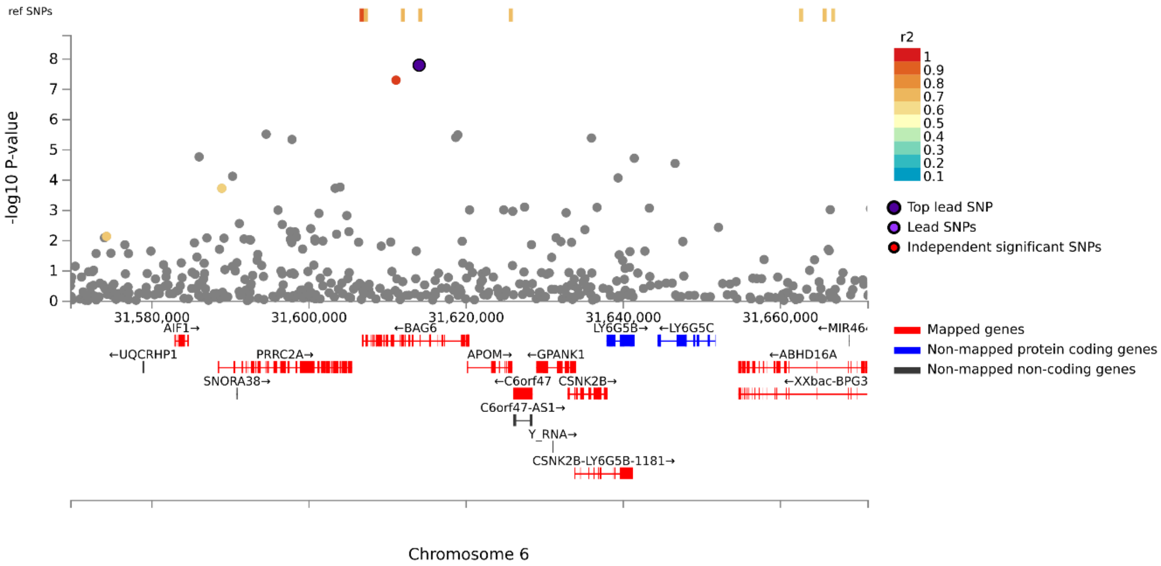

Supplementary Figure 26: Regional Manhattan Plot. rs2242656 (cross-ancestry meta-analysis).

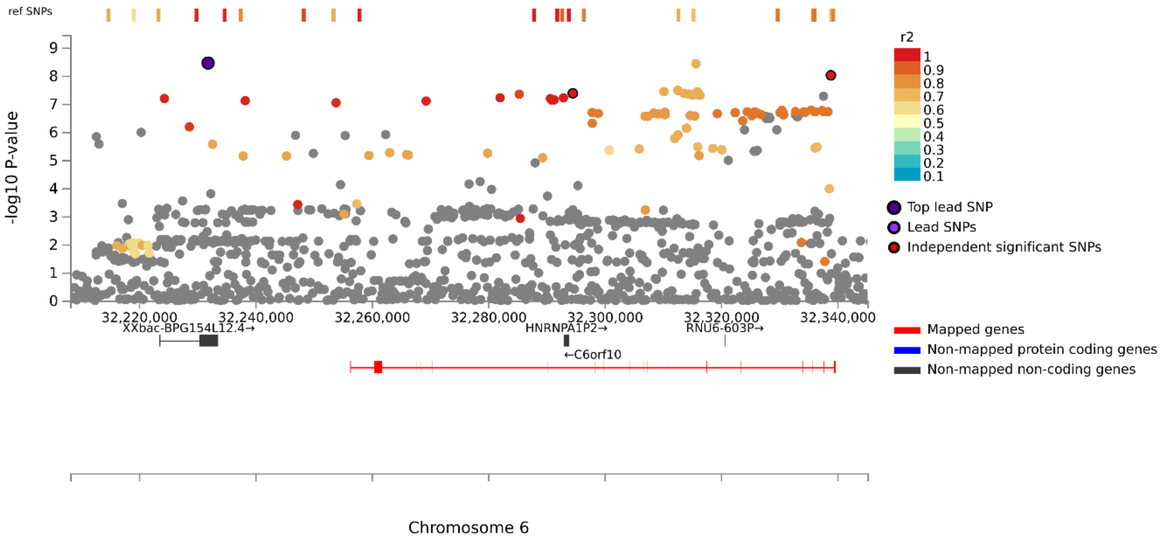

Supplementary Figure 27: **Regional Manhattan Plot.** rs9279546 (cross-ancestry meta-analysis) [note: *TSBP1* is also known as C6orf10].

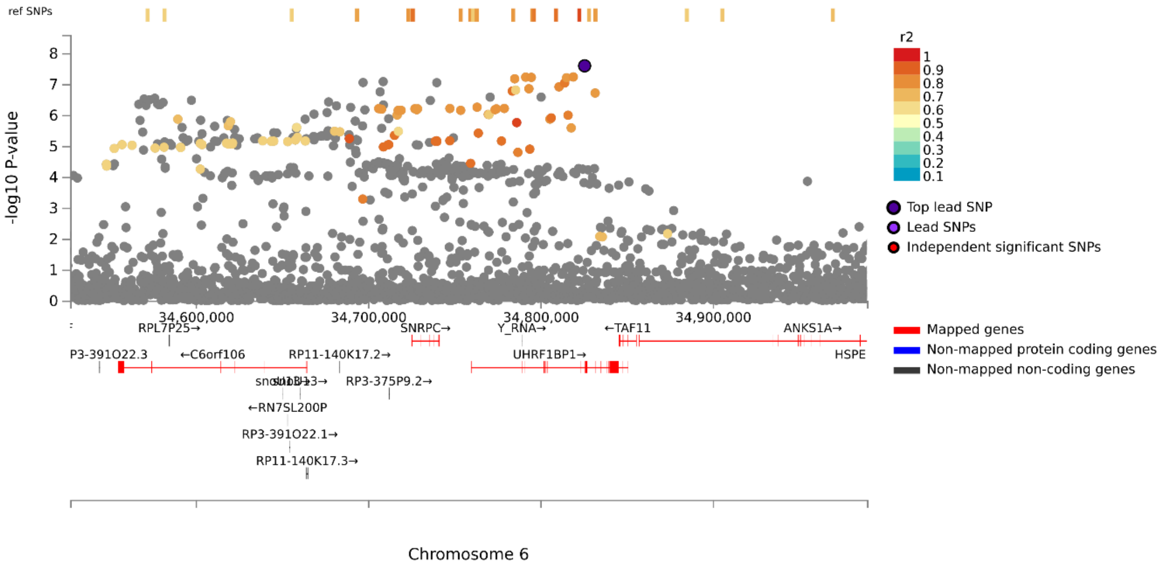

Supplementary Figure 28: Regional Manhattan Plot. rs16894959 (cross-ancestry meta-analysis).

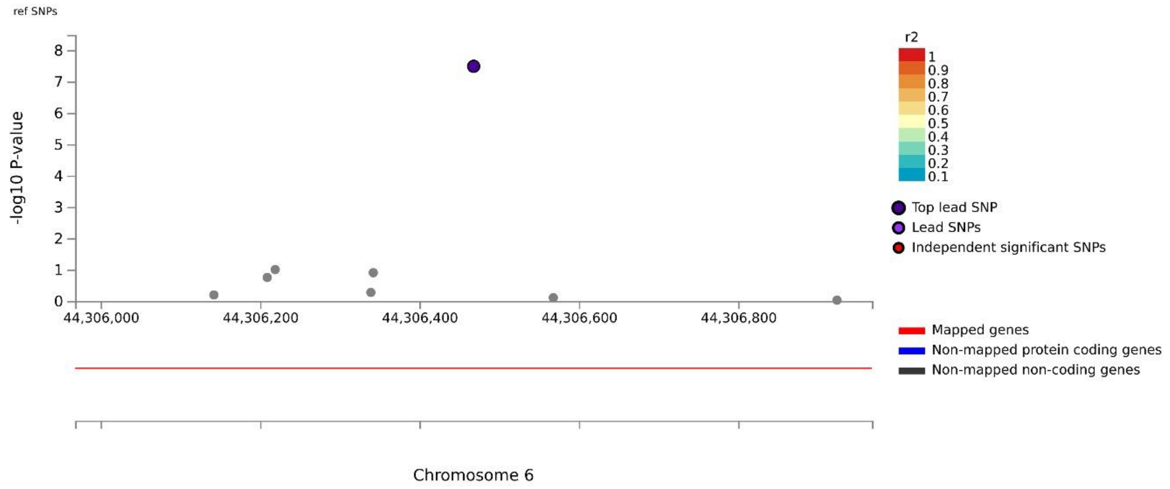

Supplementary Figure 29: Regional Manhattan Plot. rs186798404 (cross-ancestry meta-analysis).

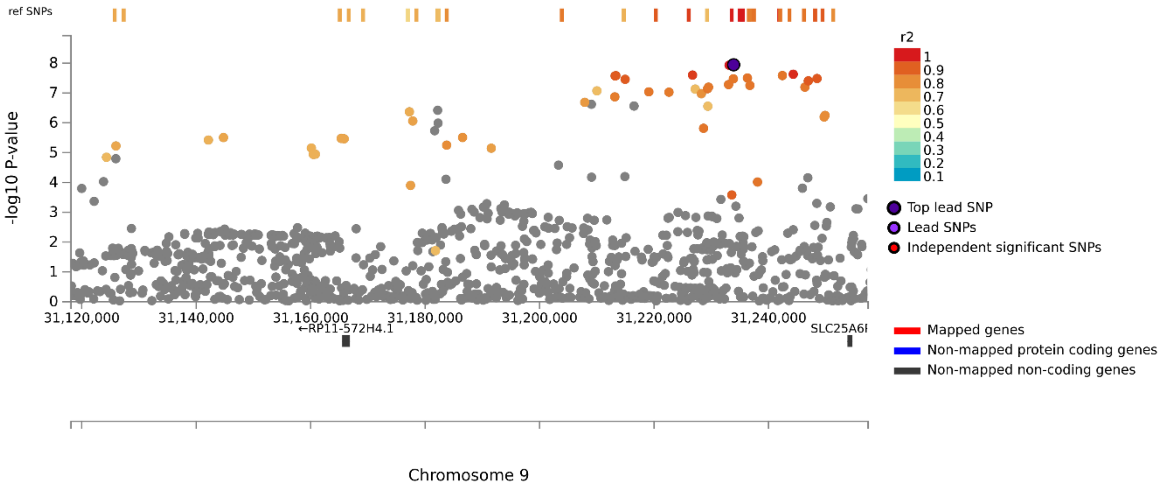

Supplementary Figure 30: **Regional Manhattan Plot.** rs12555516 (cross-ancestry meta-analysis).

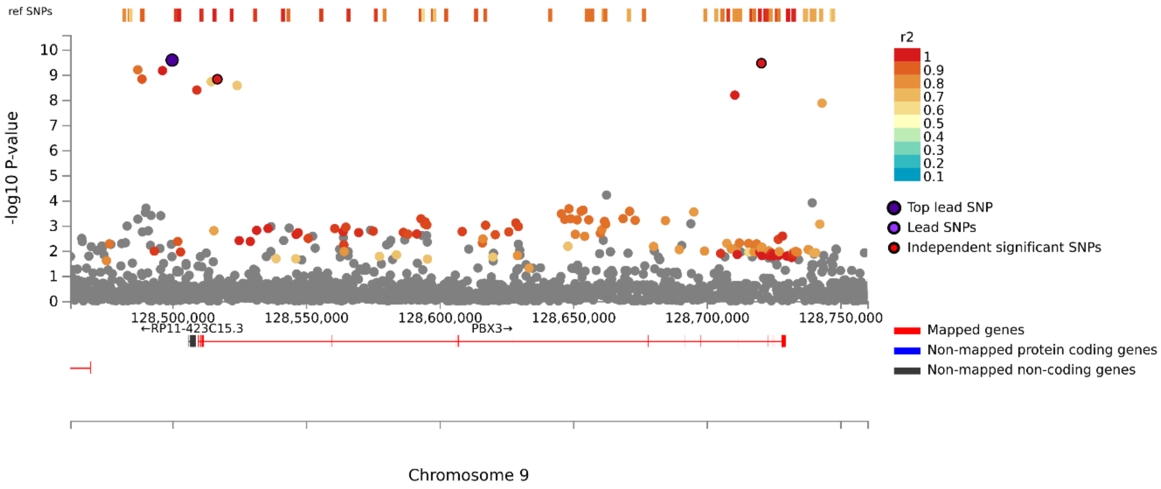

Supplementary Figure 31: **Regional Manhattan Plot.** rs10819064 (cross-ancestry meta-analysis).

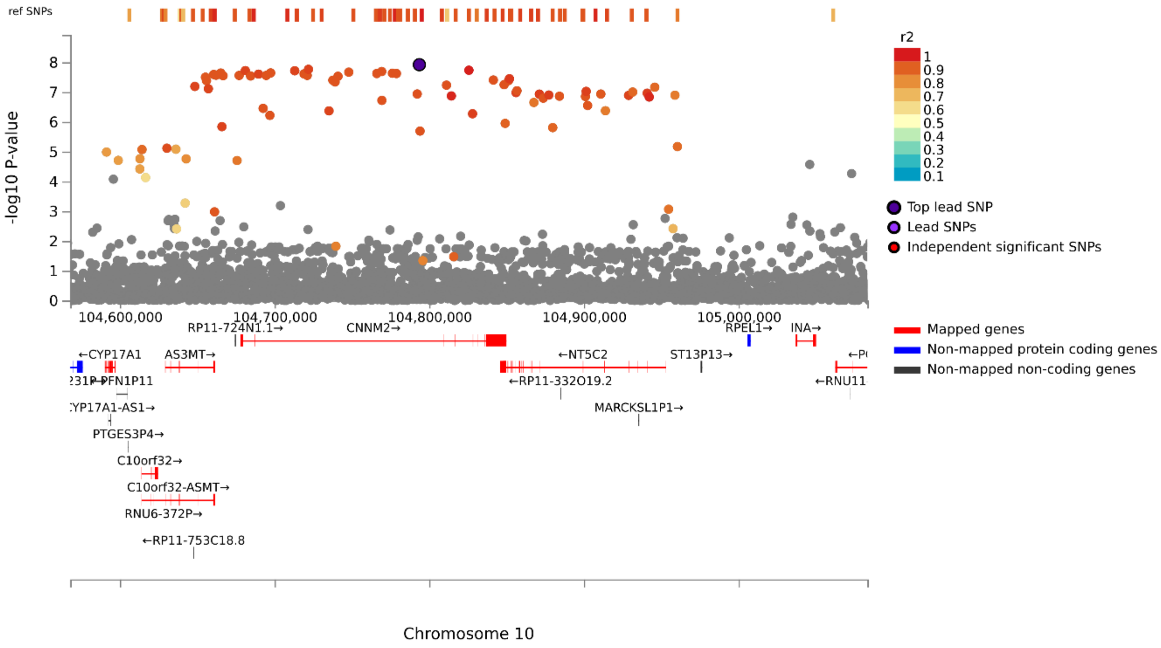

Supplementary Figure 32: Regional Manhattan Plot. rs75970938 (cross-ancestry meta-analysis).

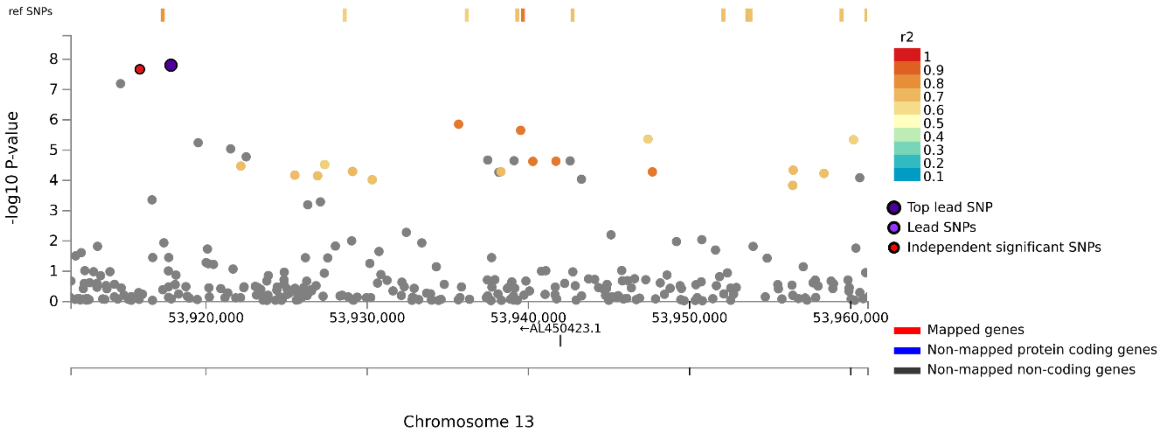

Supplementary Figure 33: Regional Manhattan Plot. rs9536401 (cross-ancestry meta-analysis).

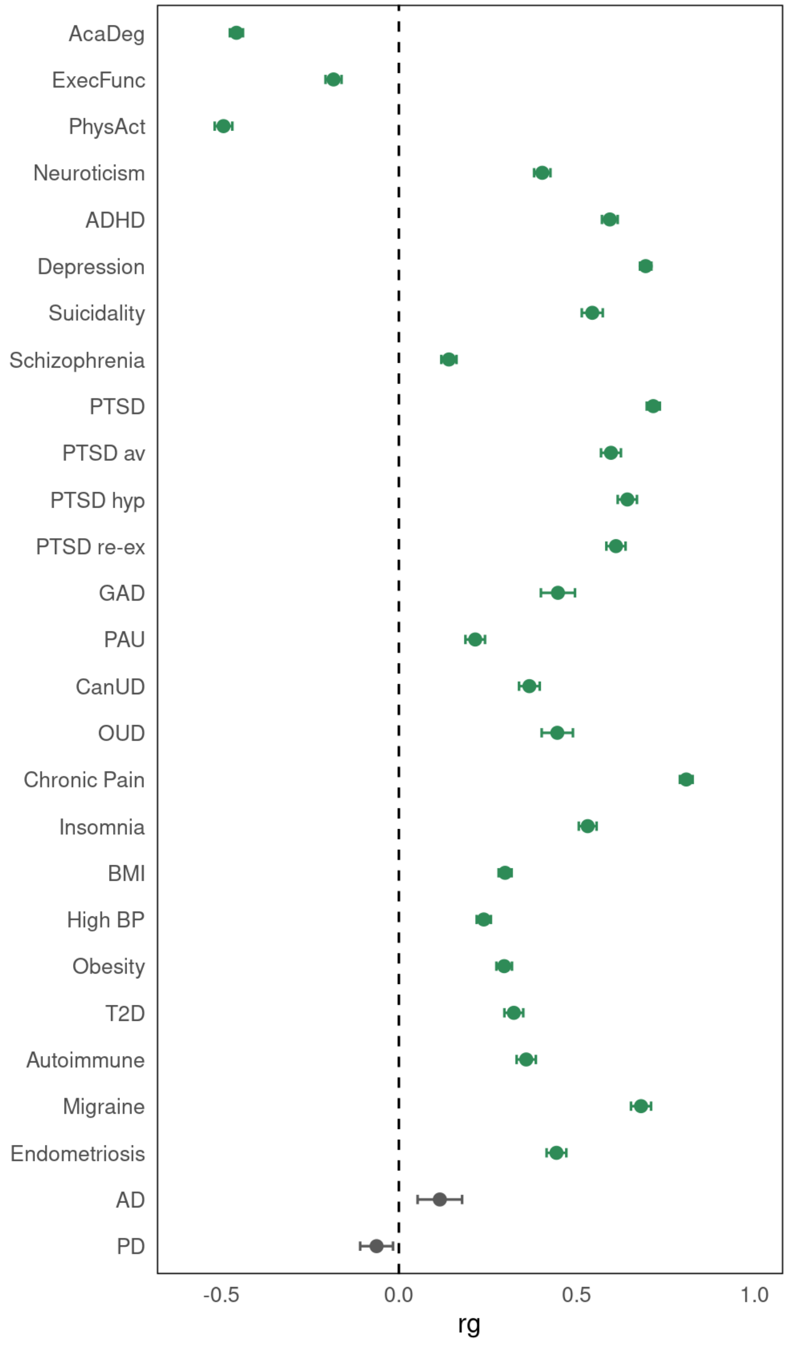

Supplementary Figure 34: Fibromyalgia MTAG Genetic Correlations. Genetic correlations between fibromyalgia MTAG and 27 traits of interest in EUR. Error bars represent standard error [AcaDeg: academic degree; ExecFunc: executive functioning; PhysAct: physical activity; ADHD: attention-deficit/hyperactivity disorder; PTSD: post-traumatic stress disorder; av: avoidance; hyp: hyperarousal; re-ex: re-experiencing; GAD: generalized anxiety disorder; PAU: problematic alcohol use; CanUD: cannabis use disorder; OUD: opioid use disorder; BMI: body mass index; BP: blood pressure; T2D: type 2 diabetes; AD: Alzheimer's disease; PD: Parkinson's disease]. Source data are provided as a Source Data file.

Supplementary acknowledgements

VA Million Veteran Program

Core Acknowledgements for Publications

June 2026

MVP Program Office

- Sumitra Muralidhar, Ph.D., Program Director
  US Department of Veterans Affairs, 810 Vermont Avenue NW, Washington, DC 20420
- Jennifer Moser, Ph.D., Associate Director, Scientific Programs
  US Department of Veterans Affairs, 810 Vermont Avenue NW, Washington, DC 20420
- Jennifer E. Deen, B.S., Associate Director, Cohort & Public Relations
  US Department of Veterans Affairs, 810 Vermont Avenue NW, Washington, DC 20420

MVP Steering Committee

- Co-Chair: J. Michael Gaziano, M.D., M.P.H.
  VA Boston Healthcare System, 150 S. Huntington Avenue, Boston, MA 021
- Co-Chair: Dave Oslin, M.D.
  Philadelphia VA Medical Center, 3900 Woodland Avenue, Philadelphia, PA 19104
- Sumitra Muralidhar, Ph.D., Ex-Officio
  US Department of Veterans Affairs, 810 Vermont Avenue NW, Washington, DC 20420
- Drew Helmer, M.D., M.S.
  Michael E. DeBakey VA Medical Center, 2002 Holcombe Boulevard, Houston, TX 77030
- Adriana Hung, M.D., M.P.H.
  VA Tennessee Valley Healthcare System, 1310 24th Avenue, South Nashville, TN 37212
- Philip S. Tsao, Ph.D.
  VA Palo Alto Health Care System, 3801 Miranda Avenue, Palo Alto, CA 94304
- Deepak Voora, M.D.
  Durham VA Medical Center, 508 Fulton Street, Durham, NC 27705

MVP Co-Principal Investigators

- J. Michael Gaziano, M.D., M.P.H.
  VA Boston Healthcare System, 150 S. Huntington Avenue, Boston, MA 02130
- Philip S. Tsao, Ph.D.
  VA Palo Alto Health Care System, 3801 Miranda Avenue, Palo Alto, CA 94304

MVP Core Operations

- Jessica V. Brewer, M.P.H., Director, MVP Cohort Operations
  VA Boston Healthcare System, 150 S. Huntington Avenue, Boston, MA 02130

- Kelly Cho, M.P.H, Ph.D., Director, MVP Phenomics
  VA Boston Healthcare System, 150 S. Huntington Avenue, Boston, MA 02130
- Lori Churby, B.S., Director, MVP Regulatory Affairs
  VA Palo Alto Health Care System, 3801 Miranda Avenue, Palo Alto, CA 94304
- Yonghui Jia, Ph.D., Director, VA Central Biorepository

VA Boston Healthcare System, 150 S. Huntington Avenue, Boston, MA 02130

- Jacob T. Kean, Ph.D., Acting Director, VA Informatics and Computing Infrastructure (VINCI) VA Salt Lake City Health Care System, 500 Foothill Drive, Salt Lake City, UT 84148
- Saiju Pyarajan Ph.D., Director, Data and Computational Sciences
  VA Boston Healthcare System, 150 S. Huntington Avenue, Boston, MA 02130
- Robert Ringer, Pharm.D., Director, VA Albuquerque Central Biorepository
  New Mexico VA Health Care System, 1501 San Pedro Drive SE, Albuquerque, NM 87108
- Luis E. Selva, Ph.D., Director, MVP Biorepository Coordination
  VA Boston Healthcare System, 150 S. Huntington Avenue, Boston, MA 02130
- Shahpoor (Alex) Shayan, M.S., Director, MVP PRE Informatics
  VA Boston Healthcare System, 150 S. Huntington Avenue, Boston, MA 02130
- Brady Stephens, M.S., Principal Investigator, MVP Information Center
  Canandaigua VA Medical Center, 400 Fort Hill Avenue, Canandaigua, NY 14424
- Stacey B. Whitbourne, Ph.D., Director, MVP Cohort Development and Management VA Boston Healthcare System, 150 S. Huntington Avenue, Boston, MA 02130
