# Supplementary material for: The genetics of fibromyalgia and its relationships to psychiatric and medical traits
Source: Nat Commun. 2026 Jul 28;17:6248. doi: 10.1038/s41467-026-75256-6 (PMC13415558; doi:10.1038/s41467-026-75256-6)
Supplement: Supplementary file 2 — Description of Additional Supplementary Files [file 41467_2026_75256_MOESM2_ESM.pdf]

Description Of Additional Supplementary Data Files

SUPPLEMENTARY DATA 1: Preliminary analysis: intra-cohort number of subjects and heritability

SUPPLEMENTARY DATA 2: preliminary analysis: intra-cohort genetic correlations (rg)

SUPPLEMENTARY DATA 3: Gene-based analysis (MAGMA) in EUR ancestry. Significant values after FDR correction are in bold.

SUPPLEMENTARY DATA 4: Gene-based analysis (MAGMA) in AFR ancestry. Significant values after FDR correction are in bold.

SUPPLEMENTARY DATA 5: Gene-based analysis (MAGMA) in AMR ancestry. Significant values after FDR correction are in bold.

SUPPLEMENTARY DATA 6: Gene-based analysis (MAGMA) in cross-ancestry meta-analysis. Significant values after FDR correction are written in bold letterts.

SUPPLEMENTARY DATA 7: Heritability estimates in the individual and meta-analyzed EUR cohorts (LDSC)

SUPPLEMENTARY DATA 8: Inter-cohort genetic correlations in EUR (LDSC)

SUPPLEMENTARY DATA 9: Genetic correlations between fibromyalgia and other traits (LDSC)

SUPPLEMENTARY DATA 10: Lead SNPs in pain-leveraged fibromyalgia MTAG in subjects of EUR genetic ancestry - a comparison with GWAS results in EUR

SUPPLEMENTARY DATA 11: Fibromyalgia MTAG leveraged by pain and MDD: lead SNPs

SUPPLEMENTARY DATA 12: Mendelian randomization (MR; using MRlap) results with fibromyalgia as exposure (p-value threshold: 1xE-05)

SUPPLEMENTARY DATA 13: Mendelian randomization (MR; using MRlap) results with fibromyalgia as outcome (p-value threshold: 1xE-05)

SUPPLEMENTARY DATA 14: Mendelian randomization (MR; using MRlap) results with fibromyalgia as exposure (p-value threshold: 1xE-08)

SUPPLEMENTARY DATA 15: Mendelian randomization (MR; using MRlap) results with fibromyalgia as outcome (p-value threshold: 1xE-08)

SUPPLEMENTARY DATA 16: Mendelian Randomization (MR): Two-sample MR

SUPPLEMENTARY DATA 17: Mendelian Randomization (MR): Horizontal pleiotropy test

SUPPLEMENTARY DATA 18:	Significant results of the local genetic correlations analysis (LAVA)
SUPPLEMENTARY DATA 19:	TWAS in EUR (FUSION)
SUPPLEMENTARY DATA 20:	Summary-based Mendelian Randomization (SMR) results
SUPPLEMENTARY DATA 21:	gSEM: confirmatory factor analysis (CFA)
SUPPLEMENTARY DATA 22:	Blood type distribution in UKBB participants
